# Long‐Term Imaging of Wound Angiogenesis with Large Scale Optoacoustic Microscopy

**DOI:** 10.1002/advs.202004226

**Published:** 2021-05-02

**Authors:** Johannes Rebling, Maya Ben‐Yehuda Greenwald, Mateusz Wietecha, Sabine Werner, Daniel Razansky

**Affiliations:** ^1^ Institute for Biomedical Engineering and Institute of Pharmacology and Toxicology Faculty of Medicine University of Zurich Zurich 8057 Switzerland; ^2^ Institute for Biomedical Engineering Department of Information Technology and Electrical Engineering ETH Zurich Zurich 8093 Switzerland; ^3^ Institute of Molecular Health Sciences Department of Biology ETH Zurich Zurich 8093 Switzerland

**Keywords:** intravital, intravital microscopy, microcirculation, photoacoustic, skin, sola cutis se reficientis, vascularization

## Abstract

Wound healing is a well‐coordinated process, necessitating efficient formation of new blood vessels. Vascularization defects are therefore a major risk factor for chronic, non‐healing wounds. The dynamics of mammalian tissue revascularization, vessel maturation, and remodeling remain poorly understood due to lack of suitable in vivo imaging tools. A label‐free large‐scale optoacoustic microscopy (LSOM) approach is developed for rapid, non‐invasive, volumetric imaging of tissue regeneration over large areas spanning up to 50 mm with a depth penetration of 1.5 mm. Vascular networks in dorsal mouse skin and full‐thickness excisional wounds are imaged with capillary resolution during the course of healing, revealing previously undocumented views of the angiogenesis process in an unperturbed wound environment. Development of an automatic analysis framework enables the identification of key features of wound angiogenesis, including vessel length, diameter, tortuosity, and angular alignment. The approach offers a versatile tool for preclinical research in tissue engineering and regenerative medicine, empowering label‐free, longitudinal, high‐throughput, and quantitative studies of the microcirculation in processes associated with normal and impaired vascular remodeling, and analysis of vascular responses to pharmacological interventions in vivo.

## Graphical abstract

Large‐scale optoacoustic microscopy is presented for long‐term imaging of vascular dynamics during wound healing in an unperturbed in vivo environment. The new method offers a versatile tool for preclinical research in tissue engineering and regenerative medicine, empowering label‐free, noninvasive, longitudinal, high‐throughput, and quantitative studies of microcirculation in processes associated with vascular remodeling, and analysis of vascular responses to pharmacological interventions.

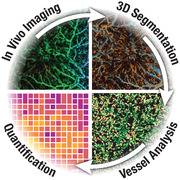

## 1 Introduction

Skin reacts to injury via a well‐coordinated healing process, which follows four overlapping phases, namely hemostasis, inflammation, proliferation, and remodeling. The skin microvasculature plays a central role in each regeneration phase, as it controls blood flow to the site of injury. This allows distribution of oxygen and nutrients and also mediates the inflammatory response and the formation of new tissue.^[^ ^1^, ^2^ ^]^ Deficiencies in wound vascularization are a main driving force of pathological healing and frequently result in the development of chronic ulcers, which pose an enormous social and economic challenge.^[^ ^1^, ^3^, ^4^, ^5^ ^]^ Accurate evaluation of wound healing and tissue regeneration is thus paramount for devising and testing optimal treatment strategies, and non‐invasive in vivo imaging provides particularly important information.

Despite skin wounds being superficial and thus easily accessible, visualization of the microvasculature network in mammalian wounds remains challenging. Therefore, translucent animals, such as zebrafish, have recently been used for long‐term imaging of the wound vasculature, which provided important insight into vessel dynamics and allowed quantification of different vessel parameters.^[^ ^6^ ^]^ Existing microscopic ex vivo imaging technologies for mouse tissues are limited by their acute nature, making extended duration studies impossible.^[^ ^7^ ^]^ State‐of‐the‐art intravital microscopy (IVM) techniques, such as confocal and two‐photon microscopy (CFM,TPM) or optical coherence tomography (OCT), can deliver high‐resolution images to visualize angiogenesis in the living skin, but are limited by the need for contrast agents and by restricted depth‐of‐focus (DOF) and generally small field‐of‐view (FOVs), hindering investigations of entire wounds and surrounding neovasculature.^[^ ^2^, ^8^, ^9^, ^10^ ^]^ Dorsal skin fold chambers overcome some of these limitations, but require highly invasive surgical implantation.^[^ ^11^ ^]^ More recently, laser speckle, ultrasound (US), and magnetic resonance imaging have enabled longitudinal wound imaging studies, but lack the resolution and specificity required to image neovascularization by fine capillaries, thus missing an essential component of wound angiogenesis.^[^ ^12^, ^13^, ^14^ ^]^ Optoacoustic (OA) imaging, based on the generation of broadband US signals following the absorption of pulsed laser excitation, provides several advantages over conventional IVM techniques, including high (scalable) resolution and penetration depth as well as label‐free sensing of spectrally‐distinct functional and molecular tissue contrast, primarily stemming from strong optical absorption of hemoglobin in functional vessels. When operated in acoustic‐resolution (mesoscopic) regime, the achievable resolution in the 25–100 µm range is adequate for high‐resolution imaging of the human skin,^[^ ^15^, ^16^ ^]^ yet does not permit visualizing the capillary network remodeling in mice. Microscopic OA imaging can instead be done in the optical‐resolution mode by focusing the excitation light spot.^[^ ^17^ ^]^ Several preclinical imaging studies have focused on imaging neovasculature and tumor angiogenesis in the murine ear and brain, similar to TPM and OCT imaging.^[^ ^18^, ^19^ ^]^ However, wounds in the dorsal skin areas, which are more relevant from the biological and clinical points of view, remain a difficult target to image with the existing scanning OA microscopy systems due to the strong optical scattering in dorsal skin, irregular wound topology, challenging combination between the high (capillary) resolution and large (centimeter‐scale) field of view, as well as breathing‐related motion artifacts. At present, no intravital imaging technique exists to enable biologically relevant longitudinal observations of microvascular remodeling during wound healing in murine models.

Here, we developed a new imaging pipeline based on large‐scale optoacoustic microscopy (LSOM) tailored for intravital, non‐invasive, longitudinal imaging of intact and wounded opaque murine skin with superb resolution and imaging speed, which allows for a quantitative analysis of vascular network alterations during wound healing.

## 2 Results

### 2.1 The Dorsal Wound Healing Imaging Setup

**Figure 1:**
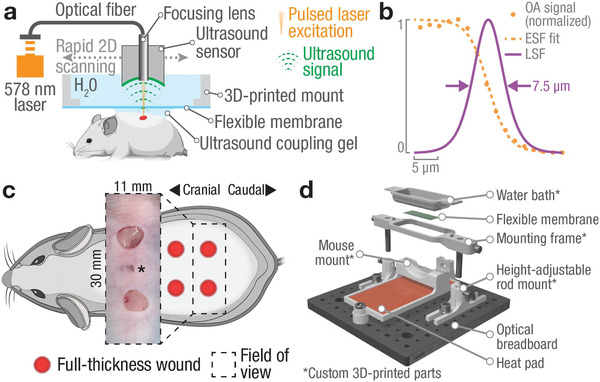
The large‐scale optoacoustic microscopy (LSOM) system and 3D‐printed mount for non‐invasive, label‐free, longitudinal monitoring of wound healing in the dorsal skin. a) Short‐pulsed laser light (578 nm) is focused through an ultrasound sensor into the skin, where it generates ultrasound waves following local optical absorption. The sensor is rapidly scanned over the dorsal skin to generate volumetric, high‐resolution images. b) Resolution characterization using an aluminum edge target showcases capillary‐level resolution of 7.5 µm measured as full‐width‐at‐half‐maximum (FWHM) of the line spread function (LSF). Orange dots – measured OA signal, dashed orange line – edge spread function (ESF) as logistic fit to OA data, solid purple line – LSF obtained by numerical differentiation of ESF. See also Experimental Section. c) Full‐thickness 5 mm excisional wounds in the dorsal skin of SKH1 mice were created. Inset showing freshly excised wounds (* indicates location of black marker used to match wound locations to pre‐wound imaging area). d) A 3D‐printed, custom‐made dorsal imaging mount stabilizes the mouse skin during imaging while providing a flat 11 × 30 mm² imaging area (see also Figure S3 and Video S1, Supporting Information).

We first designed the imaging setup to overcome the challenges of imaging large‐scale vascular networks in the murine dorsal skin (**Figure** **1**a; and Figure S1, Supporting Information). The LSOM imaging system used nanosecond laser pulses (wavelength 578 nm), which are focused onto the skin surface using an optical fiber with a focusing lens attached directly to the distal fiber tip. We used white or nude mice for our studies, since hemoglobin is the strongest absorbing tissue chromophore in these animals, while absorption by melanin is inhibiting vascular imaging in pigmented mice (e.g., C57BL/6 mice).^[^ ^20^ ^]^ The laser pulses are locally absorbed in the blood vessels, thus generating broadband US signals that are recorded using a coaxially and confocally aligned ultrawideband US sensor. A lateral resolution of 7.5 µm was achieved by using a single‐mode optical fiber and a new focusing lens design with low numerical aperture (NA = 0.05, Figure 1b and Experimental Section). The low numerical aperture enables volumetric imaging with an extended DOF of 1.5 mm in tissue. The improved resolution was crucial to resolve fine capillary networks formed during wound healing, while the high DOF aided with imaging the rough topology of the dorsal skin. We created four full‐thickness excisional wounds (5 mm diameter) in the dorsal skin of white CD‐1 and of SKH1 hairless mice (Figure 1c and Experimental Section). This model allowed visualization of angiogenesis, vascular remodeling, and regression at different days post wounding (dpw).

To enable non‐invasive imaging while maintaining a leveled and stable skin surface throughout the imaging session, we engineered and manufactured a custom, 3D‐printed dorsal imaging mount (DIM) (Figure 1d) using a low‐cost fused filament fabrication 3D‐printer (see Experimental Section; and Figures S2 and S3 and Videos S1 and S2, and 3D PDF, Supporting Information). A removable insert in the DIM acted as a water bath, which ensured acoustic coupling between skin and US sensor. An imaging window in the insert was covered with a transparent, flexible membrane, which allowed transmission of the excitation light and the generated OA responses. The underside of the DIM slightly compressed the skin, while an anatomically formed foam‐padded counterpart supported the mouse from below. Overall, an 11 mm x 30 mm x 1.5 mm volume of the dorsal skin was made available for LSOM imaging with a lateral (*x/y*) resolution of 7.5 µm and an axial (*z*) resolution of 35 µm. Additionally, a custom‐made, regulated heat pad below the mouse ensured appropriate maintenance of the mouse body temperature during anesthesia.^[^ ^52^ ^]^ The DIM thus provided an imaging platform, which combined all functions required for non‐invasive in vivo imaging without compromising the normal skin physiology.

### 2.2 High‐Resolution and Large‐Scale Imaging of Healthy and Wounded Dorsal Skin

**Figure 2:**
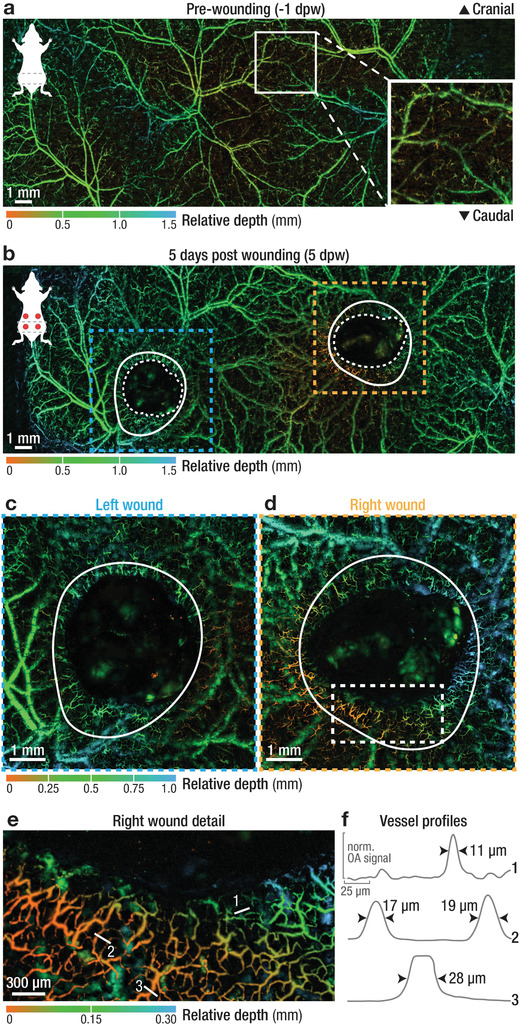
Multi‐scale imaging of intact and wounded dorsal skin. a) Depth‐encoded, large‐scale image of intact dorsal skin revealing an intricate vascular network. Insert showing superficial arterioles, venules, and capillaries supplied by deeper cutaneous vessels. b) The same area was imaged 5 days after the introduction of full‐thickness wounds, with the wounds already partially contracted and with a tortuous capillary network forming near the wound border. Blue and orange dashed lines – location of high‐resolution images shown in c and d; white solid lines mark the edge of the wound at the day of wounding (see Figure S9, Supporting Information); white dashed line marks the border of revascularization. c,d) High‐resolution recordings of the left and right wounds, as indicated in (b), showing a ring of newly formed, tortuous capillary vessels around the wound margin. e) Superficial vessels sprout directly adjacent to the wound margin and toward the wound center. White lines indicate location of vessel profiles in (f). f) Profiles of selected vessels numbered in (e), showing a relative uniform size distribution of the vascular network in the range of 10–30 µm. See Table S1, Supporting Information, for a full overview of all scan parameters and Figure S5, Supporting Information, for corresponding gross photographs.

We first used the imaging system to acquire large‐scale, high‐resolution in vivo images of the intact dorsal skin of a CD‐1 mouse. Two volumetric images were recorded approximately 4 weeks apart to showcase the non‐invasiveness and long‐term repeatability of the protocol (Figure S4, Supporting Informaion). Imaging of CD‐1 mice required skin depilation, because light penetration and acoustic coupling were otherwise suboptimal, thus reducing the image quality. To avoid repeated hair removal, we used SKH1 mice for the remaining parts of this study. The lack of hair and skin pigmentation as well as an intact immune system make SKH1 mice ideal for LSOM imaging of the wound healing process.^[^ ^21^ ^]^ We then recorded large‐scale volumetric images (11 × 30 × 1.5 mm³, 10 µm step size) of dorsal skin vasculature in 8‐week‐old female SKH1 mice (**Figure** **2**a). The observed gross cutaneous vascular network is comprised of artery–vein pairs bilaterally arising from the deep circumflex iliac arteries, similar to structures observed in skin‐flap preparations.^[^ ^22^ ^]^ The mostly parallel running artery–vein pairs branch into smaller, interconnected vessels connecting left and right aspects of the dorsal skin.^[^ ^23^ ^]^ Those smaller vessels supply superficial and highly tortuous capillaries (see insert Figure 2a), which cover the entire dorsal skin. The overall vascular morphology corresponded well with the previously reported anatomy of healthy skin of 4–6 week‐old BALB/c mice and is similar to vascular networks in the murine ear.^[^ ^24^, ^25^ ^]^

We then investigated the formation of new blood vessels in full‐thickness excisional caudal wounds at 5 dpw (Figure 2b). The large cutaneous vascular network spanning the entire mouse back was well reproduced when compared to unwounded skin (Figure 2a). Two large areas were filled with a clot and later with granulation tissue (GT), which resulted in a lack of vessel signal, clearly indicating the wound locations. The formation of GT correlates with wound closure, which involves a combination of re‐epithelialization and contraction.^[^ ^26^ ^]^ This was evident when comparing the original wound border (solid white lines) with the contracting wound edge (white dashed lines) (Figure 2b, and Figure S5, Supporting Information). To better visualize the newly formed vasculature, we recorded high‐resolution images (7 × 7 mm² FOV, 5 µm step size) centered on both the left and right wound (blue and orange dashed lines, Figure 2c,d). During early wound healing, we observed a ring of tortuous capillaries around the wound margin, clearly visible in both wounds.^[^ ^27^ ^]^ This dense, highly irregular, capillary network appeared near the wound border (2 × 3 mm² FOV, 2 µm step size) (Figure 2d,e). Here, superficial arterioles and venules (orange vessels, profiles 2 and 3 in Figure 2f, size > 15 µm) sprouted into even smaller terminal arterioles and capillaries (green to blue vessels, profile 1 in Figure 2f, size < 15 µm) adjacent to the wound margin.

### 2.3 Long‐Term Imaging of Intact and Wounded Dorsal Skin

**Figure 3:**
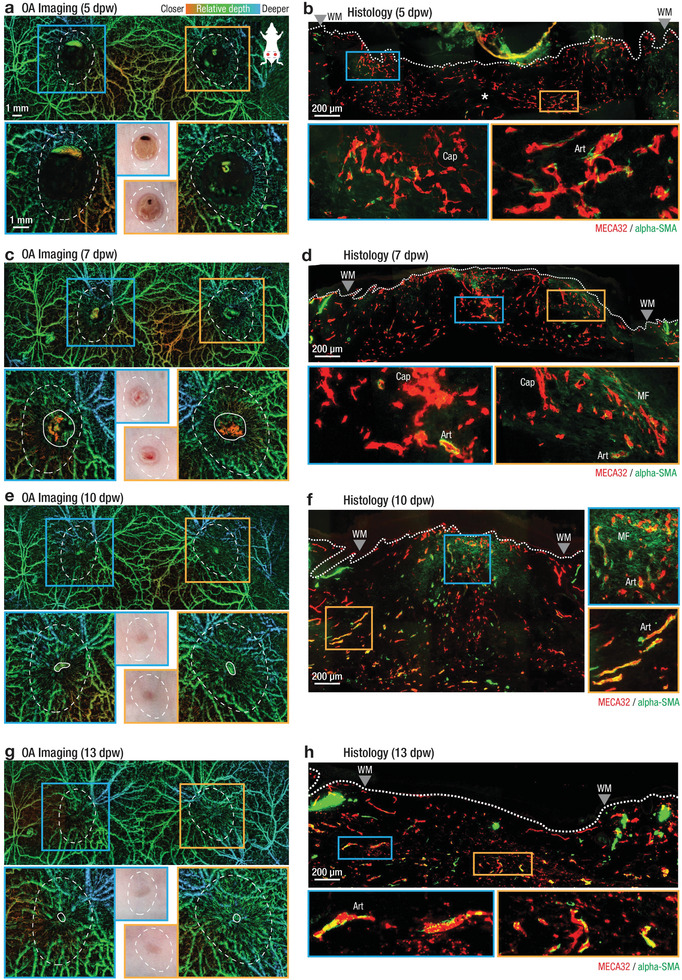
Observation of large‐scale angiogenesis and remodeling during cutaneous wound healing over a two‐week period using non‐invasive, label free LSOM and the corresponding histology in SKH1 mice. a,c,e,g) In vivo LSOM images of wounded dorsal skin at 5,7,10, and 13 dpw. Top panels provide a large‐scale overview of the dorsal vascular network, while bottom panels show a high‐resolution scan concentrating on the left (blue box and outline) and right (orange box and outline) wound regions. Middle inserts show gross appearance of wounds as digitally photographed. White dashed lines indicate approximate location of original wound area (see Figure S9, Supporting Information). White solid lines indicate border of wound edge vasculature. Images are color‐coded for relative vessel depth. b,d,f,h) Side view of excisional wounds at 5, 7, 10, and 13 dpw. Tissues were sectioned and stained using antibodies against MECA‐32 for endothelial cells (red) and *α*‐SMA for vascular smooth muscle cells and myofibroblasts (green); mature vessels show MECA‐32/*α*‐SMA co‐localization (yellow). Myofibroblasts are *α*‐SMA positive and MECA‐32 negative (faint green in (d,f). At 5 dpw, angiogenesis is limited to small caliber vessels near the original wound margins (a, white dashed lines), where histologically highly branched, sprouting, superficial capillaries (b, blue box), deep arterioles near the wound edge (b, orange box) and little vasculature in the wound center (b, white asterisk) were observed. Wound vasculature begins aligning toward the wound center at 7 dpw (c), also visible histologically as maturing, aligned arterioles at the wound edge surrounded by vascular smooth muscle cells (d, orange box). Macroscopic wound closure is complete at 10 dpw (e, photo inserts) with vascular remodeling ongoing, visible as more aligned mature vessels in both in vivo LSOM images (e) and histologically (f, orange box). At 13 dpw, angiogenesis is complete, with the entire wound closed (g), and histologically with mature vessels visible at the wound edge (h, blue box) and in the wound center (h, orange box). See Figure S10, Supporting Information, for additional wound staining and Figure S11, Supporting Information, for separate MECA‐32 and *α*‐SMA channel images. Art – arterioles; Cap – capillaries; WM – wound margins, MF – myofibroblasts.

We next performed non‐invasive LSOM imaging over the course of two weeks in SKH1 mice (Figure S6, Supporting Information). The images were recorded one day prior (−1 dpw) to injury and at different time points of the healing phase (**Figure** **3**a–d; and Figure S7, Supporting Information). LSOM imaging of the dorsal skin ex vivo was also performed for each time point (Figures S7 and S8, Supporting Information). The images consistently manifested high quality and contrast during the entire observation period, in contrast to previous TPM studies where image quality significantly deteriorated past 8 dpw.^[^ ^28^ ^]^ We recorded the superficial wound appearance using a miniature bright‐field microscope while utilizing the custom DIM to flatten the skin and wound surface, which allowed for non‐invasive tracking of wound closure and contraction (photo inserts Figure 3a,c,e,g; and Figure S9, Supporting Information). Following the in vivo imaging sessions, mice were sacrificed and cranial and caudal wound sections were used for histological analysis and immunofluorescence staining for markers of blood endothelial cells (Meca‐32) and vascular smooth muscle cells/myofibroblasts (*α*‐smooth muscle actin or *α*‐SMA) (Figure 3b,d,f,h; and Figures S10 and S11, Supporting Information). Histologically, intact skin of the SKH1 mice presented with typical features of this mouse strain, including dermal cysts and enlarged sebaceous glands (Figures S10 and S11, Supporting Information). Vessel analysis showed superficial capillaries (green box), deeper paired venules and arterioles (purple box), and larger arterioles of the hypodermis (orange box) (Figures S10 and S11, Supporting Information), thus confirming the in vivo non‐invasive LSOM observations in 2D (Figure 2a). The wounds gradually healed as expected, with early formation of an eschar and complete re‐epithelialization at around 5 dpw, combined with gradual contraction and maturation of the GT (Figures S10 and S11, Supporting Information). Top‐viewed wound closure and wound‐edge vascularization were quantified as the ratio of grossly re‐epithelialized and re‐vascularized areas compared to the initial wound areas (0 dpw) (see Experimental Section; Figures S12 and S13, Supporting Information).

Progressive wound closure was observed macroscopically (Figure 3g; and Figure S12, Supporting Information; and Experimental Section) and confirmed histologically (Figures S10 and S11, Supporting Information). The onset of angiogenesis was visible in the OA images at 5 dpw (Figure 3a) with an average wound‐edge re‐vascularization of 40% (Figure S13, Supporting Information). Wound edge vessels sprouted as they invaded the wound tissue, visible as irregular, small caliber vessels inside the wound region (white dashed lines). Histological analysis revealed a similar pattern of sprouting vasculature (Figure 3b), with highly branched superficial capillaries (Cap, blue box), deep arterioles (Art, orange box) near the wound edge and with little vasculature present in the wound center (white asterisk). A thick eschar above the wound center may have interfered with reliable LSOM imaging of this area at 5 dpw (Figures S10 and S11, Supporting Information). Wound edge vasculature was clearly visible in the LSOM images in all wounds at 7 dpw after gentle removal of the eschar (Figure 3c; and Figure S13, Supporting Information), with most vessels within the original wound area oriented toward the center. These vessels were also visible as maturing, aligned arterioles in the wound edge surrounded by *α*‐SMA‐expressing vascular smooth muscle cells (Art, blue box, co‐localization of both Meca‐32 and *α*‐SMA, yellow) in the histological images (Figure 3d). In contrast to the clearly visible, aligned vessels at the wound border, LSOM signals that originated from the center of granulation tissue appeared as bright, but unstructured areas (inside white solid lines, Figure 3c; and Figure S13, Supporting Information). Histological analysis suggested that these LSOM signals are indicative of a dense capillary network forming under the highly scattering tissue at the wound center (blue box Figure 3f). Top‐viewed wound edge vascularization had progressed considerably at 7 dpw, with an average of 72% of the wound area being vascularized (Figure S13, Supporting Information).

All wounds were completely closed (re‐epithelialized) at 10 dpw (Figure 3e,g) (Figure S12, Supporting Information), which was confirmed histologically (Figures S10 and S11, Supporting Information) and was also apparent in the LSOM images (Figure 3e,g). Wound edge re‐vascularization was almost complete at 10 dpw, with a 96% average re‐vascularization, manifesting as small, dense, and highly oriented vessels within the original wound area (white dashed lines) (Figure 3c). At 13 dpw, all wounds were completely re‐vascularized, with 99% of wound areas exhibiting a dense vascular network. Histological analysis again supported these in vivo observations, with dense arterioles appearing in the wound center (Figure 3f, blue box) and highly aligned arterioles at its edges (Figure 3f, orange box) at 10 dpw. Also, highly aligned mature vessels are visible at the wound edge (Figure 3h, blue box), while the vascular density is lower at its center (Figure 3h, orange box) at 13 dpw, suggesting the onset of vessel regression. As expected, re‐vascularization closely followed the healing process, with the highest vascularization rate observed at 5.5 dpw (Figures S12 and S13, Supporting Information).

Our imaging protocol also allowed for the observation of deeper arterioles and venules in the interstitial connective tissue layer just below the *panniculus carnosus* muscle. These larger vessels were located below the superficial, smaller vessels, spanning the entire dorsal skin (Figure 2a; and Figure S7, Supporting Information). Upon wounding, the formation of an eschar and later of a dense GT/scar tissue partially obstructed these deep vessels (Figure 3a,c,e,g). Since the imaging contrast chiefly stems from the light absorption by hemoglobin in perfused vessels, we were further able to create large‐scale, ex vivo images of the skin's flipside, which resulted in an unobstructed view of the intact deep vessel morphology (Figures S7 and S8, Supporting Information). Notably, only moderate vessel remodeling has taken place when comparing the vascular networks in the pre‐wounded (−1 dpw; and Figure S7, Supporting Information) and healed (13 dpw, Figure 3g) skin. Large vascular plexuses were found both left and right of the centerline, appearing almost unaltered in their morphology after healing (white arrow heads; and Figure S7, Supporting Information). The left wound apparently severed a larger, looping vessel, which was able to recover and appeared perfused at 13 dpw (white arrow). In contrast, a large vessel crossing the right wound region was not restored during the healing process (white asterisk). All ex vivo images showed excellent contrast for larger vessel structures, but small superficial vessels were not visible (Figure S8, Supporting Information).

### 2.4 Automatic LSOM‐Based Volumetric Vessel Analysis

**Figure 4:**
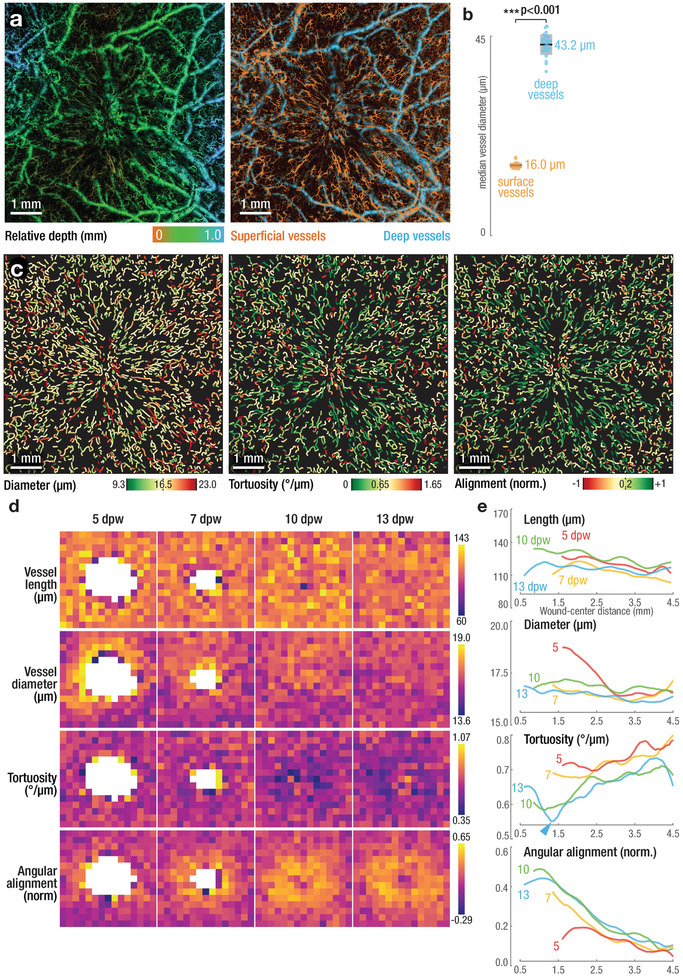
Automatic, high throughout, and quantitative analysis of vessel and network morphology based on volumetric LSOM imaging enables entirely non‐invasive measures of wound re‐vascularization. a) Depth‐resolved, volumetric LSOM datasets (left panel) were automatically segmented into superficial and deep vasculature (orange and blue, respectively, in the right panel) for subsequent analysis. b) Comparison of vessel sizes reveals significant differences between shallow vessels in the dermis when compared to arterioles and venules situated deeper in the interstitial connective tissue (*n* = 28 wounds; Student's *t*‐test, *p* = 4.8 × 10^−51^). Dots represent median vessel diameter of individual wounds; black lines represent mean values; gray boxes represent 95% confidence intervals. c) Automatic vessel analysis extracts vessel parameters for thousands of individual vessels per wound (average of 1547 ± 93 vessels). Exemplary visualizations of vessel diameter (left panel, mean diameter = 16.5 µm), tortuosity (middle panel, mean tortuosity = 0.65° µm^−1^), and vessel alignment (right panel, mean alignment = 0.2; higher values indicate vessel alignment toward wound center). d,e) Vessel parameters (length, diameter, tortuosity, and angular alignment) for all wounds over the time course of healing (*n* = 10, 8, 6, and 4 wounds for 5, 7, 10, and 13 dpw, respectively). Heat maps (d) empower a qualitative assessment of aggregated spatial vascular alterations, while representation relative to their distance from the wound center (e) allows evaluating changes over the wound time course. Wound healing appears to be fueled by changes in tortuosity and alignment, rather than length and diameter of the vessels. Heat map size 6 mm x 6 mm, binning size 400 µm x 400 µm. See Experimental Section for definition of tortuosity and alignment. Panels (a) and (c) show exemplary data of a wound at 13 dpw.

Our data indicates that a strong growth and remodeling of the superficial capillaries occurs during the healing process, while larger vascular structures appear relatively unaffected.^[^ ^24^ ^]^ In contrast to planar fluorescence imaging and other 2D imaging techniques, we utilized the volumetric 3D nature of the LSOM datasets to segment the superficial vessels and distinguish them from the large deeper‐situated vessels. This allowed for a quantitative analysis of vessel‐network parameters over the course of healing without obscuration by strong background signals from large vessels. The automatic extraction of quantitative vessel parameters such as vessel length, diameter, tortuosity, or direction is an essential step toward a reliable analysis of vascular remodeling in healing wounds.^[^ ^2^ ^]^ We developed a segmentation algorithm based on the separation of superficial small capillaries and deeper arterioles and venules (right panel, **Figure** **4**a; Experimental Section; and Figure S14, Supporting Information), which was facilitated via our recently developed vessel detection method.^[^ ^29^ ^]^ A significant difference in the median vessel diameters was found between the segmented superficial (16.0 µm mean diameter; 15.3–17.8 µm range) and deep (43.2 µm mean diameter; 37.1–47.3 µm range) vessel volumes (Figure 4b).

As wound angiogenesis is driven by capillary sprouting, we focused our quantitative analysis on small superficial vessels. Key parameters such as location, length, diameter, tortuosity, and angle were determined for each vessel and for all wounds, as shown for a single wound at 13 dpw (Figure 4c). Long straight vessels with a low tortuosity of 0.65° µm^−1^ or less (green and yellow vessels, central penal, Figure 4c) were oriented toward the wound center in a radial pattern. To examine the relationship between vessel parameters and wound closure, we normalized the wound locations with respect to their center and created heat maps for functional vessel length, diameter, tortuosity, and angular alignment aggregated for all imaged wounds and timepoints (Figure 4d). Similarly, the same vessel parameters were further plotted as a function of distance from the wound center for all post‐wounding timepoints (Figure 4e). Both vessel length and diameter changed only insignificantly over the course of healing, with lengths in the 110–140 µm range and diameters from 16–17 µm for all dpw (first and second row in Figure 4d; 1st and 2nd row in Figure 4e). A notable exception was observed at 5 dpw, where a clear increase in the vessel diameter was visible at the wound edge, approximately 1.5 mm from its center, likely indicating active vessel sprouting and edema (Figure 4e, 2nd row, red solid line). In contrast, larger changes were observed for the vascular tortuosity and angular alignment. The former was strongly reduced near the wound center in the original wound area from 7 dpw onwards, while the tortuosity values remained as low as 0.35° µm^−1^ at 10 dpw (Figure 4d, 3rd row). At 10 dpw, vessels were becoming straighter (i.e., having a lower tortuosity) when approaching the wound center (Figure 4e, 3rd row, green line). This overall trend continued for wounds at 13 dpw, where the tortuosity was reduced even further (1.4 mm from the wound) (Figure 4e, 3rd row, blue line, and blue arrowhead). Angular alignment, defined as tendency of the individual vessels to align toward the wound center, consistently increased over time, with vessels also becoming increasingly aligned when approaching the wound center (Figure 4d, 4th row; Figure 4e, 4th row). The only exception to this trend were vessels near the wound border at 5 dpw (Figure 4e, 4th row, red solid line). Their alignment was reduced for distances of 1.5–2.5 mm from the center, again likely indicating active sprouting. This observation in combination with the increased vessel size at 5 dpw suggested that the previously reported ring of larger caliber vessels was only present at the early stages of wound healing with vessel diameter normalizing between 5 and 7 dpw.^[^ ^30^ ^]^ Based on our findings, angiogenesis in wound healing is mainly associated with changes in the vascular network, that is, changes in vessel tortuosity and direction, rather than alterations in length and diameter of the individual vessels.

## 3 Discussion and Conclusions

We developed a non‐invasive, and label free imaging and analysis pipeline that enables, for the first time, long‐term visualization of blood vessels in non‐injured and wounded dorsal mouse skin. Our LSOM method, based on volumetric imaging of optical absorption, was used to monitor vascular and structural characteristics coupled to the various phases of wound healing and tissue regeneration at capillary‐level resolution. The technique overcomes several key challenges faced by existing methodologies as it allows the direct, repeated in vivo visualization of vascular networks and their remodeling with high resolution and over long periods. This is in stark contrast to microscopic ex vivo analyses employing histology or microCT angiography, where animals are sacrificed at each time point.^[^ ^31^, ^32^ ^]^ Intravital fluorescence microscopy methods are capable of in vivo longitudinal imaging, but are limited to relatively small and superficial areas of the wound, further necessitating extrinsic labeling.

The newly established mount for dorsal skin imaging is a valuable tool for the analysis of microcirculation, representing an attractive solution for numerous challenges faced in vessel imaging. Much like the dorsal skinfold chamber (DSC), the DIM allows repeatable and reproducible access to large skin areas that are easy to image.^[^ ^30^ ^]^ In this way, the need for time‐consuming depth scanning is eliminated, as both non‐injured (Figure 2a) and wounded (Figure 2b–e) skin is effectively flattened to achieve surface height variations below 1.5 mm. Unlike the surgically implanted DSC, no invasive procedures are required. Neither the microvasculature nor the complex wound healing process are thus affected by surgical implantation and the required post‐operative care. Observation periods are also not limited when using the removable DIM, compared to few weeks after which conventional DSCs lose their function.^[^ ^33^ ^]^ Our approach further provides an imaging area which is orders of magnitude larger when compared to DSC and other conventional imaging windows (>300 mm² for DIM versus <3 mm² for DSC).^[^ ^30^ ^]^ The DIM is simple, inexpensive to manufacture and assemble, as it is entirely comprised of 3D printed or of‐the‐shelf components, compared to the costly titanium‐based DSC solutions. The DIM can furthermore be used for other imaging modalities such as ultrasound or fluorescence microscopy. Care must be taken when placing the mice in the dorsal imaging mount in order to achieve a reproducible FOV for imaging due to the loose interstitial connective tissue and thus highly mobile skin. Images or photographs from previous imaging sessions may aid with an accurate placement. If precise co‐registration of the FOV over multiple imaging timepoints is required, micro‐tattoos or prominent vessel locations (e.g., plexus) could be used to align the vascular networks.

Cutaneous wounds heal in an orderly progression, with neovascularization and vessel regression having a fundamental impact on the outcome.^[^ ^34^ ^]^ Robust angiogenesis initiates approximately 3 to 4 dpw when capillaries “move into the wound space as a unit.*”* ^[^ ^35^ ^]^ Previous intravital microscopy studies referred to the distinct capillary pattern formed during wound healing as “sola cutis se reficientis,” due to its resemblance of a sun.^[^ ^30^ ^]^ Sorg et al. describe a ring of circular vessels directly at the wound edge supplied by outer, radially oriented, and more directional vessels. A similar morphology was revealed by microCT angiography.^[^ ^32^ ^]^ Here, we detected vessels of a larger caliber entering the wound periphery, running toward its center, then terminating at the edge, resulting in a dense microvascular network. These complex vascular network patterns were visualized on a much larger scale than was so far possible (Figures 2 and 3). This provides a significant advantage over previous studies, where only small fractions of the wound area were observed, or other ex vivo investigations where the need for a contrast or casting agent perfusion permitted “only arterioles [to be] studied, but not large veins or capillaries.”^[^ ^30^, ^32^ ^]^

Another key distinguishing factor of our method is its fully non‐invasive and label‐free nature. Once the mouse was positioned in the DIM and under the LSOM imaging head, repeated scanning in multiple locations was achieved without additional positioning and height adjustments. This allowed for precise determination of the area of interest via quick overview scans followed by increasingly detailed scans targeting specific regions. The combination of the flexible DIM with the large FOV and scalable resolution thus created a high‐throughput imaging system. For instance, the recording session for Figure 2b–e, including mouse anesthesia, handling, positioning, repeated imaging, and mouse recovery required less than 30 min altogether (see Table S1, Supporting Information). In comparison, intravital microscopy and previously reported OA microscopy systems covering comparable FOVs only record a single image during that time.^[^ ^24^, ^36^ ^]^ In addition to the technical advance, our approach strongly contributes to animal welfare, allowing reduction of the number of animals due to the possibility of consecutive imaging and the avoidance of invasive procedures.

The automatic extraction of quantitative vessel parameters is an essential step to reliably analyze and compare vascular remodeling in healing wounds.^[^ ^2^ ^]^ Our algorithm, in combination with the volumetric nature of our imaging approach, was then utilized to quantify changes of the vascular morphology over time and with high resolution. A quantitative assessment of the vessel parameters was hampered in previous studies due to the lack in FOV, resolution and/or capacity to resolve both small and large vessels. Yet, the overall trends of existence of larger caliber vessels near the wound center at 5 dpw as well as the reduced tortuosity and increased vascular alignment at 7 dpw is in agreement with previous studies.^[^ ^30^, ^32^ ^]^

The LSOM system offers an optimal trade‐off between resolution, FOV, and depth‐penetration for many skin imaging applications. The effective imaging depth exceeding 1 mm competes well with established techniques such as TPM or CFM and is sufficient to image both superficial capillaries and the underlying venules and arteries without additional depth‐scanning. The lateral resolution of 7.5 µm is adequeate for distinguishing individual vessels down to the level of single capillaries, with typical diameters below 10 µm.^[^ ^37^ ^]^ However, as the vessel diameter approaches the lateral resolution limit of the system, deconvolution‐approaches are required in order to avoid overestimation of the true vessel diameter.^[^ ^38^ ^]^ Our technique could furthermore be used in combination with either TPM (to achieve higher resolution) or other acoustic‐resolution OA systems (for deeper penetration), while utilizing the same DIM. As OA imaging is based purely on optical absorption contrast, imaging of the commonly used C57BL/6 mice is difficult with the presented approach due to the strong absorption of melanin pigments. The use of white or hairless mice mitigates this constraint. The single wavelength excitation utilized in the present work could potentially be extended to multiple wavelengths, thus enabling label‐free imaging of functional parameters such as oxygen saturation and blood flow.^[^ ^39^ ^]^ While providing a powerful tool for studies on murine model organisms, clinical translation of the optically‐resolved LSOM method is hindered by the limited depth penetration when employing focused light excitation beam. Acoustically‐resolved OA mesoscopy^[^ ^16^, ^40^ ^]^ or tomography^[^ ^41^, ^42^, ^43^ ^]^ techniques are therefore better suited for skin and wound imaging in humans.

Since LSOM does not entail the use of contrast agents or surgical interventions, the effect of different compounds on the vasculature can be studied in an unperturbed physiological environment, without affecting post‐imaging histological validation. Both topical application and injection of pharmaceutical substances is possible, facilitating the study of experimental agents that affect angiogenesis.^[^ ^44^, ^45^ ^]^ Our approach may further reduce the need for histological analysis. In this way, the required mouse numbers can be reduced while further accelerating progress in the field by delivering a more comprehensive picture of the vascular remodeling process in 3D. The unique features offered by our method lend themselves to applications beyond dorsal skin imaging. The flexible epi‐illumination design could be used to image ex vivo samples or other areas such as the ear, skull, brain, paw, or tail in murine models. Future in vivo studies could unravel how healing dynamics are influenced, for example, by wound location or contraction. Ultimately, LSOM could assist in the validation of novel therapeutic strategies to promote or suppress vascularization and analysis of re‐vascularization in other wound models, such as splinted wounds. It could also help to determine how angiogenesis is affected in healing‐impaired diabetic mice and elucidate aspects of vascular remodeling in skin grafting and artificial tissue constructs or in tumors.^[^ ^24^, ^46^, ^47^, ^48^ ^]^

## 4 Experimental Section

##### The Large‐Scale Optoacoustic Microscopy (LSOM) Setup

A comprehensive diagram of the optical and electrical components comprising the large‐scale optoacoustic microscopy (LSOM) system is shown in Figure S1, Supporting Information. The system was designed for dorsal skin imaging by improving imaging speed, resolution, and FOV as compared with a previously reported implementation.^[^ ^29^ ^]^ A diode‐pumped solid‐state Nd:YVO4 laser (IS80‐2‐L, EdgeWave, Würselen, Germany) pumping a dye laser (custom solution based on Credo, Sirah Lasertechnik, Grevenbroich, Germany) tuned to a 578 nm wavelength, created 7 ns laser pulses, which were used for OA excitation primarily targeting optical absorption by hemoglobin. The per‐pulse energy was adjusted to 1 µJ using a half‐wave plate (AHWP10M‐600, Thorlabs, Newton, NJ, USA) in combination with a polarizing beam splitter (PBS251, Thorlabs). The excitation light was cropped by an iris (SM1D12SS, Thorlabs) to match the aperture of the aspheric coupling lens (AL1225M‐A, Thorlabs) mounted in a *z*‐axis translation mount (SM1Z, Thorlabs). Approximately 4% of the beam energy was redirected by means of a beam sampler (BSF10‐A, Thorlabs) onto a photodiode (DET10A, Thorlabs). The coupling lens focused the light into a single‐mode fiber (SMF; 460HP, Nufern, East Granby, CT, USA) mounted inside a translational mount (ST1XY‐D/M, Thorlabs) with >40% coupling efficiency. The distal end of the SMF terminated in a custom made gradient‐index (GRIN) lens (GRINTECH, Jena, Germany) mounted in a central aperture of a spherically focused polyvinylidene fluoride (PVDF)‐based ultrasound sensor (Precision Acoustics, Dorchester, UK) having >100% effective bandwidth ≈35 MHz central frequency. The GRIN lens focused the 578 nm light pulses into a diffraction‐limited 7.5 µm spot at a distance of 6.5 mm. The optical focus of the GRIN lens and the acoustic focus of the transducer were aligned coaxially and confocally, which resulted in an optimized detection sensitivity. For volumetric imaging, the scan head (comprised of transducer and GRIN lens) was rapidly oscillated along the *x*‐axis by means of a fast voice‐coil stage (VCS; X‐DMQ12P‐DE52, Zaber Technologies, Vancouver, Canada) while a second linear stage (not shown; LNR50SEK1/M, Thorlabs) moved slowly and continuously along the *y*‐axis, thus sampling the imaging target in a sinusoidal pattern. Depth‐resolved OA signals (A‐scans) were recorded by the US transducer and amplified by a 24 dB low‐noise amplifier (ZFL‐500LN, Mini‐Circuits, Brooklyn, NY, USA) before being digitized by a 2‐channel, 250 MS/s, 16‐bit data acquisition (DAQ) card (M4i4420‐x8, Spectrum Systementwicklung Microelectronic, Grosshansdorf, Germany). The second DAQ channel was synchronously digitizing the photodiode signal for correction of per‐pulse laser energy fluctuations. A custom‐made, microcontroller‐based read‐out board (not shown) was used to read the VCS position in real time.^[^ ^49^ ^]^ This allowed for a position‐based trigger scheme where a second custom‐made trigger board synchronized laser excitation and data acquisition.^[^ ^16^, ^50^ ^]^ The digital raw data recorded during imaging were buffered in memory and saved for post‐processing. Post‐processing consisted of digital bandpass‐filtering (2–50 MHz, third‐order Butterworth), per‐pulse energy correction and reshaping to volumetric datasets. Volumetric projections were created via maximum‐amplitude projection (MAP) of the volume dataset and contrast limited adaptive histogram equalization (CLAHE) was applied to compress the large dynamic range of the OA signals for better visualization of small vascular structures. All images were displayed as depth‐encoded MAPs. The depth mapping was “relative”, that is, the most elevated part of the imaged skin was defined as zero depth and displayed using an orange color. Structures located deeper were color‐coded depending on their position relative to the zero‐depth. This allowed an overview of the overall skin topography while also visualizing small capillaries in front of larger arterioles and venules. The LSOM system control was executed by a custom software written in Matlab 2020a (Mathworks, Natick, MA, USA) while the custom trigger and readout board firmware were written in C/C++. A live‐preview during imaging provided instant feedback on the imaged target, displaying both a CLAHE processed and depth‐encoded MIP as well as a B‐scan (*x–z* slice) at the current scan‐head location. Data recording and post‐processing were performed on a conventional personal computer (Intel Core i7‐6800k processor, 64 GB memory, Windows 10). Video S2, Supporting Information, exemplifies the LSOM operation during scanning of a printed imaging phantom.

##### Spatial Resolution Characterization

The lateral resolution of 7.5 µm was measured by scanning a sharp edge of a strongly absorbing material (orange dots, Figure 1b), as described elsewhere.^[^ ^25^ ^]^ The ESF was found as a logistic fit of the form

(1) $$y\left(x\right)=\;\frac{1}{1+e^{-k\left(x-x_{0}\right)}}$$

to the OA data with *k* being the steepness of the curve and *x*_0_ the edge location (dashed orange line, Figure 1b). Robust, nonlinear least squares fitting based on least absolute residuals was performed using a Levenberg–Marquardt algorithm. The line‐spread function (LSF) was then calculated as the numerical gradient (∂*y*/∂*x*) of the fitted ESF and the resolution measured as the full‐width‐at‐half‐maximum (FWHM) of the normalized LSF (solid purple line, Figure 1b). The resulting 7.5 µm resolution was only slightly inferior to the 5.3 µm diffraction limit when focusing a 578 nm laser beam with NA of 0.05. The 35 µm axial resolution (along the depth dimension) was determined by the 30 MHz effective bandwidth of the US detector.^[^ ^25^ ^]^

##### Dorsal Imaging Mount (DIM)

The epi‐illumination design of the imaging LSOM system in combination with its large DOF allowed for a flexible scanning of uneven surfaces. For an initial image quality assessment, four wounds 5 dpw were imaged by placing the mouse on a simple bar to elevate its back and allow access for the freely moving scan‐head (see Figure S2a, Supporting Information). A dense capillary network surrounding the healing wound as well as larger surrounding vessels are visible in Figure S2b, Supporting Information, for a caudal wound at 5 dpw. While the overall image quality was acceptable, there was a strong tilt in the skin surface, with the top‐right area being more elevated than the bottom left area, thus creating an out of focus region despite the imaging systems’ large DOF. Additionally, strong breathing artifacts were observed, which are visible as horizontal lines in Figure S2b, Supporting Information. Both the surface tilt and the breathing artifacts were a direct result of the uneven surface topology on the mouse back. For this particular reason, intravital imaging of dorsal wounds so far required the use of implanted imaging windows, despite their obvious drawbacks such as acute surgical trauma, altered vessel morphology, and microcirculation as well as limitations for long‐term imaging studies.^[^ ^51^ ^]^

The DIM offered a completely non‐invasive, fast, cost‐effective, and easy to use alternative for large‐scale imaging of dorsal skin without the requirement for any invasive procedures. The DIM was comprised of a removable imaging window mounted onto a base for supporting the mouse (Figure 1d; Figure S3a, Supporting Information). The assembly of the dorsal imaging mount was demonstrated in Video S1, Supporting Information. The imaging window included the water bath, a flexible membrane, a mounting frame, and two metal rods (ER1, Thorlabs, USA). The base was formed by a 15 cm x 15 cm optical breadboard (MSB15/M, Thorlabs) onto which the mouse mount and the rod mounts for the imaging window were fixed. The mouse mount further contained a 50 mm x 100 mm flexible, waterproof heat‐pad (245‐528, RS Components GmbH, Frankfurt, Germany) and a digital temperature sensor (190‐4576, RS Components GmbH). The head‐pad included a custom‐made controller board, which controlled and displayed mouse and pad‐temperature in real time.^[^ ^52^ ^]^

The DIM was comprised entirely of standard or 3D‐printed parts and components, making duplication and adaptation fast, affordable, and easy. Due to the DIM's completely non‐invasive nature, observation durations were not limited by the imaging method employed; thus repeated imaging over a period of weeks or months is possible. By adapting the mounting frame to carry a standard microscopy coverslip, the DIM was made compatible with conventional CFM or TPM. Design and assembly of the DIM was performed using Autodesk Inventor Professional 2021 (Autodesk, USA). Design files as well as print ready files can be found online.^[^ ^55^ ^]^

##### Animals Handling and Wounding Experiments

Female CD‐1 and SKH1 hairless mice (8–10 weeks old; Charles River Laboratories, Germany) were housed and received food and water ad libitum. Mice were anesthetized using ketamine/xylazine or isoflurane inhalation. The back skin was shaved, further applying a depilatory cream (Veet, Reckitt Benckiser, Heidelberg, Germany). After 2 min, the hair removal cream was cleared away and the skin was cleaned with water and 70% ethanol. Four full‐thickness excisional wounds (5 mm diameter) were generated on either side of the back midline using disposable biopsy punches. Wounds were allowed to heal without dressing. Wound scabs were left intact until imaging at 5 dpw (see Figure S9, Supporting Information). After contacting with the US gel used for better OA coupling, the scabs became soft and were easy to remove. None of the animals displayed signs of discomfort or disturbances in eating or drinking habits and all wounds healed without complications. Mouse maintenance and all animal experiments had been approved by the local veterinary authorities (Kantonales Veterinäramt Zürich, Switzerland).

##### Histology

Following euthanasia of the mice, wounds were excised and either fixed with ethanol/acetic acid 95:1 v/v overnight followed by paraffin embedding or frozen in tissue‐freezing medium (Leica Microsystems, Wetzlar, Germany). Paraffin‐embedded wound sections (7 µm) from the middle of the wound were stained with hematoxylin and eosin (H&E) and imaged with a Panoramic 250 Slide Scanner (3D Histech, Budapest, Hungary).

##### Immunofluorescence

Wound cryosections (7 µm) were washed with PBS containing 0.1% Triton X‐100 followed by fixation in cold acetone. Sections were then washed and blocked with PBS containing 12% BSA and 0.1% Tween‐20 for 1 h at RT, followed by an incubation with primary antibodies. The following day, sections were incubated with the secondary Cy3‐conjugated secondary antibody and counterstained with Hoechst 33342 (Sigma, Munich, Germany). Stained sections were imaged using a Zeiss AxioImager M2 microscope with a motorized stage at 20x, magnification (Carl Zeiss Inc., Jena, Germany). Zen Pro software (Carl Zeiss) was used to control the camera and to stitch together individual photomicrographs into a complete wound image.

The antibodies used for immunofluorescence are described in **Table** **1**.

**Table 1:** Detailed specifications of the antibodies used for immunofluorescence

Antibody	Source	Dilution	Incubation conditions	Identifier
Rat anti‐Meca‐32	BD Biosciences, Franklin Lakes, NJ	1:1000	Overnight at 4 °C	Cat#553849; RRID:AB_395086
Mouse anti‐*α*‐smooth muscle actin‐FITC	Sigma	1:500	Overnight at 4 °C	Cat#F3777; RRID:AB_476977
Anti‐rat‐Cy3	Jackson ImmunoResearch, West Grove, PA	1:200	30 min at RT	Cat#715‐165‐150; RRID:AB_2340666

##### Large‐Scale Dorsal Skin Imaging

For in vivo imaging, mice were anesthetized in an induction chamber using 3% isoflurane in an oxygen/air mix. During imaging, anesthesia was maintained via a breathing mask at 2% isoflurane. Mice were placed on the regulated heat‐pad built into the DIM to maintain a body temperature of 37 °C and eye protection cream was applied (see Figure S3, Supporting Information). A fresh flexible membrane was used for each mouse and placed between the water bath and mounting frame (Figure 1d). To achieve efficient acoustic coupling between the ultrasound transducer and mice, a small amount of coupling medium (50% US gel; Aquasonic, Parker Laboratories, Fairfield, NJ, USA, and 50% PBS; Sigma) was applied to the mouse skin. The removal imaging window was then inserted and pressed down gently to flatten and stabilize the dorsal skin. Gross anatomical images of the skin were created using a digital microscope (Dino‐Lite AM7915MZT, AnMo Electronics, Taiwan) prior to LSOM imaging (Figures S5, S9, and S12, Supporting Information). The water bath mounted inside the imaging window was then filled with deionized water and the assembly was placed in the center of the FOV of the LSOM system. All liquids were heated to 37 °C prior to application. A first preview (11 mm x 30 mm FOV, 50 µm pixel/step size) provided a quick overview and allowed for an optimal positioning of the skin with respect to the acoustic and optical foci, thus attaining volumetric datasets with high resolution and high contrast. After optimizing the positioning, a large‐scale (11 mm x 30 mm FOV) scan was performed at high resolution (10 µm step size) to render the intricate details of the dorsal microvascular network. The FOV was then centered on the wound locations and another preview scan (7 mm x 7 mm FOV, 25 µm step size) was recorded. For volumetric wound imaging at capillary level, the FOV was 7 mm x 7 mm with a 5 µm lateral step size. Scan parameters and scan times are listed in Table S1, Supporting Information. All mice tolerated imaging well and recovered from anesthesia without complications. Ex vivo imaging of the excised and flipped dorsal skin (Figure S8, Supporting Information) was performed following the same procedure. The maximum laser per‐pulse energy was 1 µJ at a maximum repetition rate of 10 kHz.

##### Healing Score and Wound–Edge Vascularization

Macroscopic photographs were used to assess the progression of wound healing. For each wound, the initial wound area was marked based on the macroscopic images recorded on the day of wounding (Figure S9, Supporting Information) and compared to the apparent wound border (white dashed and solid lines, Figure S12, Supporting Information). As indicated in the insert of Figure S12, Supporting Information, a healing score was defined as the ratio of healed versus wounded area, with values close to 0 representing no healing and values close to 1 indicating full, superficial wound closure. Similarly, wound–edge vascularization was measured as the ratio of re‐vascularized area to the original wound area (Figure S13, Supporting Information). Superficial wound healing was then analyzed over time. To investigate the wound closure dynamics, a logistic fit (see Equation (1) above) of the individually measured healing/vascularization scores, was used. The superficial healing and re‐vascularization rates were calculated as the normalized numerical gradient of the logistic fit.

##### Vessel Segmentation and Analysis

Since wound healing is driven by capillary sprouting, the analysis was targeted on small, superficial vessels. As a first step, all LSOM volumes were segmented into two volumes, one containing small, superficial vessels, and the other containing larger, deeper located vessels (Figure 4b). While the DIM was capable of sufficiently flattening the skin for high‐throughput imaging, the skin surface was not entirely leveled, as indicated by the right and left areas located approximately 1 mm deeper than the center in the left panel of Figure 4a. Thus, this volume segmentation could not be done using a simple, planar surface. Thus, a volume segmentation algorithm was developed, as shown in Figure S14, Supporting Information. The LSOM datasets were projected into 2D maximum amplitude projections (MAPs) while also extracting the depth‐index of the maximum signal. A multiscale vessel enhancement was used to differentially boost signals from small and large vessel, respectively.^[^ ^53^ ^]^ Depth indices for areas containing small but not large vessels were used as support for surface fitting. A continuous surface representing the separation plane between superficial and deep vessels was then calculated in two ways. A linear interpolation provided an accurate but noisy surface while a fifth‐order polynomial surface provided a smooth separation plane. The average of the two was then used to separate the original LSOM‐rendered volume into two volumes containing superficial and deep vessels. Following the volumetric segmentation, superficial vessels were analyzed using a previously described automatic vessel segmentation and analysis algorithm (AVSA) optimized for the LSOM datasets.^[^ ^29^, ^54^ ^]^ The AVSA provided precise positions of the vessel segments (start point, end point, centerline), thus providing various morphological parameters, such as vessel length, diameter (Figure 4c, left panel), or angle. Furthermore, vessel tortuosity (angular change vs distance, Figure 4c, center panel) was measuredas well as angular alignment, namely, vessel angle deviation with respect to the wound center (Figure 4c, right panel). The large 7 mm x 7 mm FOV in combination with the high spatial resolution resulted in datasets containing thousands of vessels (average of 1547 ± 93 vessels at 5–13 dpw). Using highly‐vectorized implementations for all time‐critical steps in the algorithm, the AVSA processing step took less than 10 s per individual wound dataset when running on a conventional PC, thus enabling high‐throughput imaging and analysis. To generate the vessel‐parameter maps displayed in Figure 4d, the vessel location was first normalized with respect to the apparent wound center, determined as the center of mass of the non‐vascularized areas (solid white lines, Figure S13, Supporting Information) for each individual wound. Vessels were then grouped into 400 µm x 400 µm areas based on their position relative to the wound center. Every 400 µm x 400 µm then represented the local, average set of vessel parameters calculated for all wounds. Similarly, Figure 4e, displays the average vessel parameters found at each dpw for all wounds when visualized as a function of distance from the wound center.

## Conflict of Interest

The authors declare no conflict of interest.

## Supporting information

Supporting Information

Supplemental Video 1

Supplemental Video 2

## Data Availability

Research data are not shared.
