# Supplementary material for: Long‐Term Imaging of Wound Angiogenesis with Large Scale Optoacoustic Microscopy
Source: Adv Sci (Weinh). 2021 May 2;8(13):2004226. doi: 10.1002/advs.202004226 (PMC8261523; doi:10.1002/advs.202004226)
Supplement: Supplementary file 1 — Supporting Information [file ADVS-8-2004226-s001.pdf]

© 2021 Wiley-VCH GmbH

ADVANCED SCIENCE
Open Access

Supporting Information

for *Adv. Sci.,* DOI: 10.1002/advs.202004226

Long-Term Imaging of Wound Angiogenesis with Large Scale Optoacoustic Microscopy

Johannes Rebling, Maya Ben-Yehuda Greenwald, Mateusz Wietecha, Sabine Werner, and Daniel Razansky

Supporting Information

Long-Term Imaging of Wound Angiogenesis with Large Scale Optoacoustic Microscopy

Johannes Rebling, Maya Ben-Yehuda Greenwald, Mateusz Wietecha, Sabine Werner, and Daniel Razansky

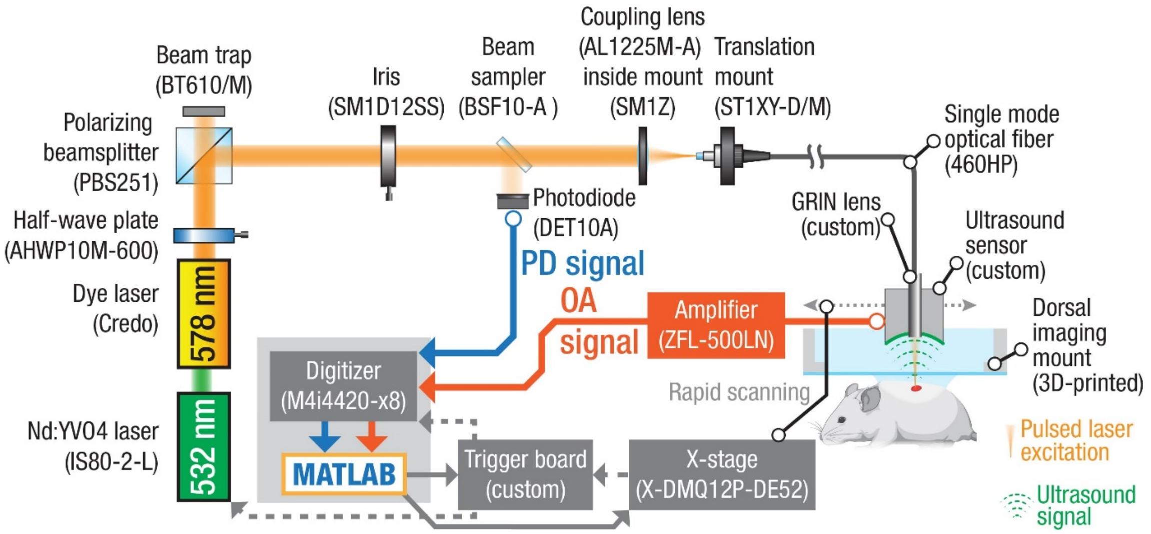

Figure S1. Detailed LSOM imaging system overview. A diode pumped, solid state laser (532 nm) pumps a dye laser tuned to 578 nm for achieving maximum endogenous contrast based on optical absorption by hemoglobin. The excitation light is guided into a single-mode optical fiber. A gradient index (GRIN) lens attached at its distal end focuses the beam into the sample, where optoacoustic (OA) signals are generated. A single-element, spherically focused polyvinylidene fluoride (PVDF) film-based transducer detects the generated OA signals which are then amplified and digitized. The transducer mount is scanned over the skin, and laser excitation and digitization are synchronized by a position-based trigger signal provided by a custom trigger board. Part numbers are shown in brackets, see online methods for details. PD – photodiode.

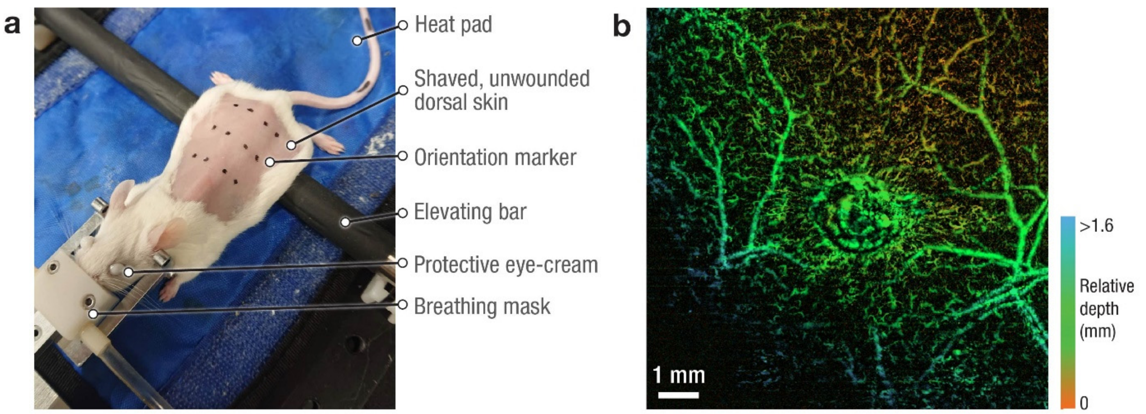

Figure S2. Suboptimal imaging of dorsal skin due to improper mounting. a Naïve approach to image dorsal wounds. **b** Resulting depth encoded LSOM image of a caudal wound at 5 dpw with strong overall tilt in the image, resulting in significant signal reduction in out-of-focus regions (bottom left).

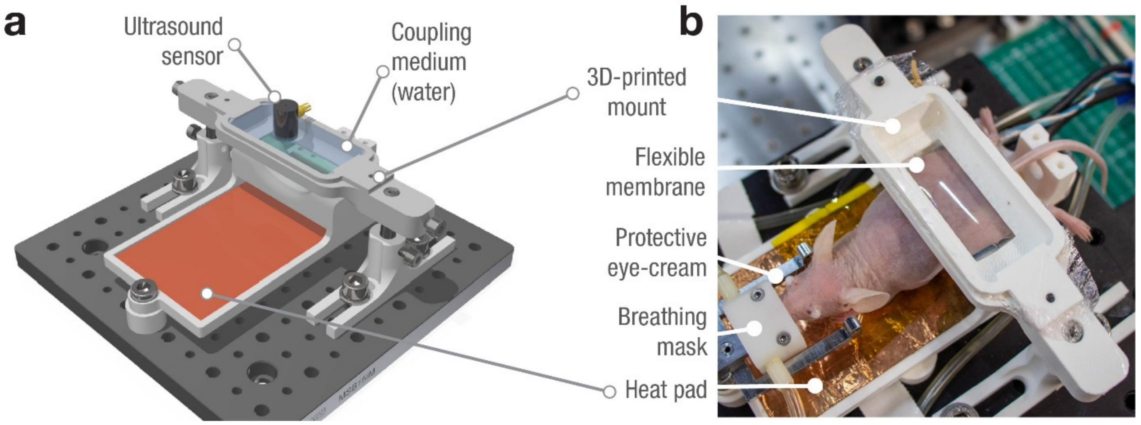

Figure S3. The dorsal imaging mount (DIM). a Rendering of the assembled mount. The majority of the assembly is comprised of 3D printed parts, making duplication and adaptation fast, affordable, and easy. The assembly is illustrated in Supplementary Video 1 and Supplementary PDF 1 and 3D design files can be wound here. **b** Assembled mount before *in vivo* imaging of a SKH1 mouse with breathing mask. The DIM applies minimal pressure to flatten the dorsal skin while not perturbing the circulation.

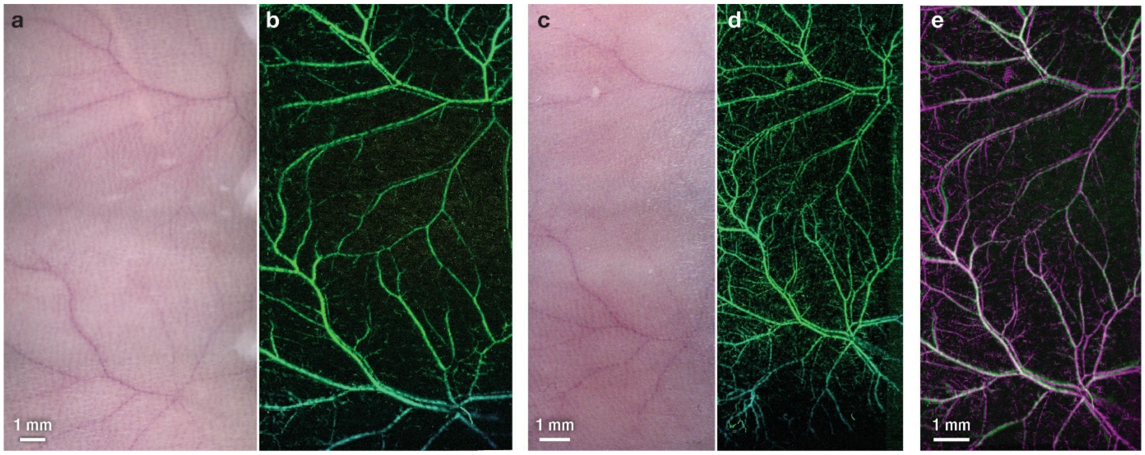

Figure S4. Long-term and large-scale LSOM imaging of CD-1 mice. a, b Gross photography and LSOM image of dorsal skin of a CD-1 mouse following hair removal on the skin. **c, d** Identical mouse imaged two weeks later following second hair removal. **e** Composite of b and d, showcasing repeatability of LSOM imaging without the need for invasive imaging windows or markers. White – vessels in both b & d, purple – vessels in d, green vessels in b.

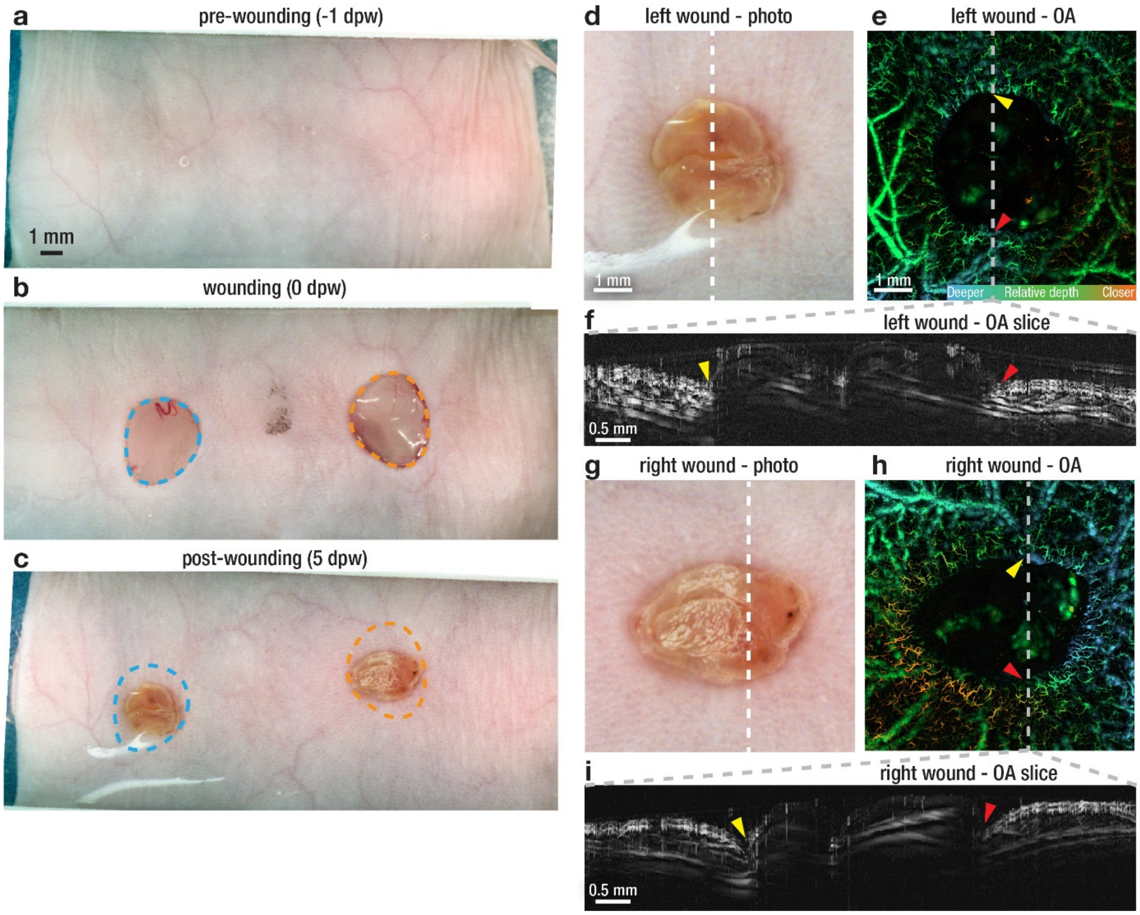

Figure S5. Gross photography of dorsal skin in SKH1 mice before and after wounding and detailed wound visualization. a Intact dorsal skin (-1 dpw). **b** Freshly wounded skin (0 dpw). **c** Partially healed and slightly contracted wound (5 dpw) corresponding to Figure 2b. Orange and blue dashed lines indicate approximate position of full wound at 0 dpw. **d-i** Detailed views of gross wound appearance (d,g) with corresponding OA image (e,h) and OA slice (f,i) for left and right wounds respectively. Magnified views of left and right wounds corresponding to Figure 2c and d respectively. White dashed lines indicate OA slice locations, red and yellow arrow hats indicate wound border.

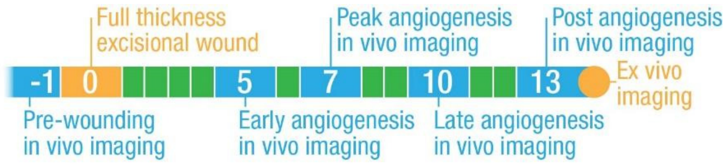

Figure S6. Wounding and imaging timeline for long-term imaging. SKH1 mice (n = 5) where imaged one day prior to wounding (-1 dpw) and/or 5, 7, 10 and 13 dpw with LSOM. At each post-wounding imaging time point (5, 7, 10 and 13 dpw) one mouse was sacrificed for additional ex-vivo imaging and histological analysis of the wounded area.

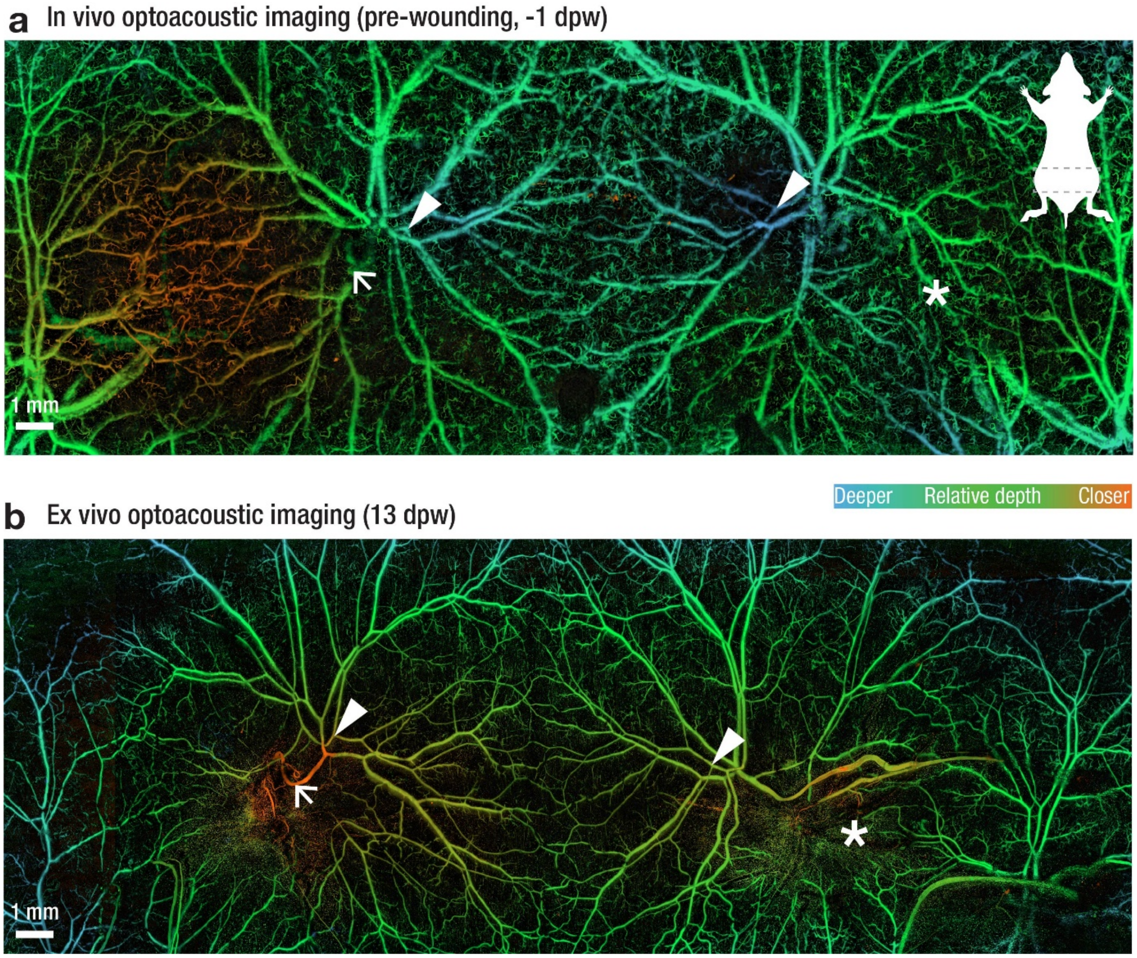

Figure S7. Comparison of large vessel morphology using *in vivo* and *ex vivo* label-free LSOM. a Pre-wounding LSOM imaging of the intact dorsal skin revealing two large vascular plexuses (white arrow heads) supplying the left and right dorsal skin. **b** *Ex vivo* LSOM imaging of the excised dorsal skin at 13 dpw enables an unobstructed view of deeper vessels located in the interstitial connective tissue and shows remodeling of some (white arrow) but not all (white asterisk) large vessels in the wounded area.

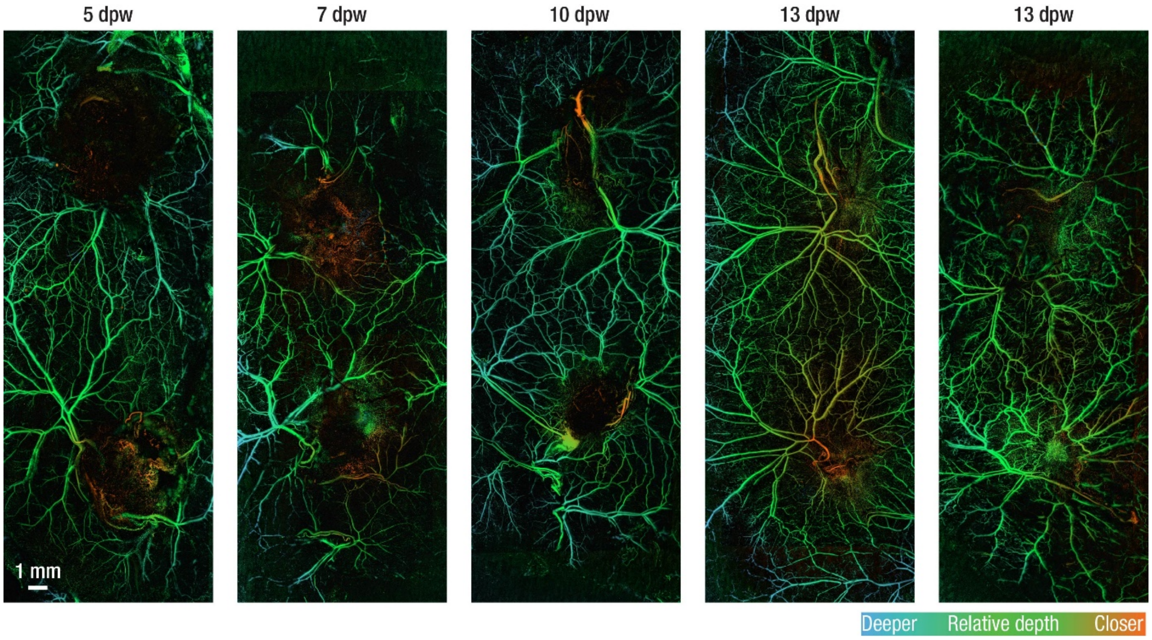

Figure S8. Label free, *ex vivo* LSOM imaging of excised dorsal skin at 5, 7, 10 and 13 dpw. Imaging was performed after excision and flipping of the dorsal skin, so that the interstitial connective tissue layer was imaged directly. Large caliber vessels forming the plexus are visualized with high contrast. Anatomically superficial (in this view deep) vessels visible in the *in vivo* images (Figure 2, 3) cannot be seen.

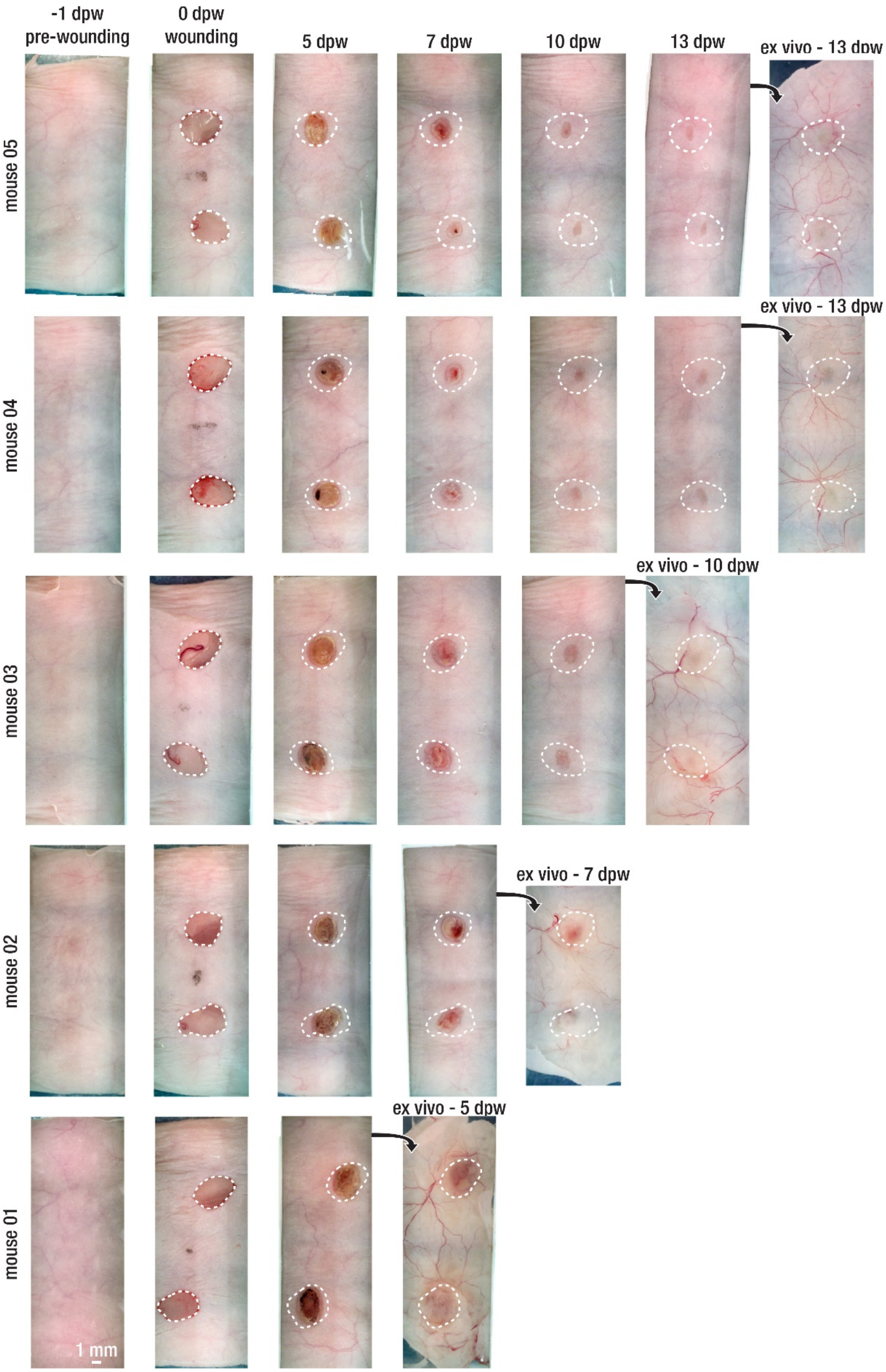

Figure S9. Gross, macroscopic photographs of dorsal skin. Panels show unwounded skin (left), wounded skin (2nd from left) and healing wounds. Right-most panels show *ex vivo* skin as imaged with LSOM (see Figure S8) and prior to histological fixation (Figure S10). White dashed lines indicate the initial wound region based 0 dpw images, manually matched to post-wound imaging time points (5-13 dpw and ex vivo).

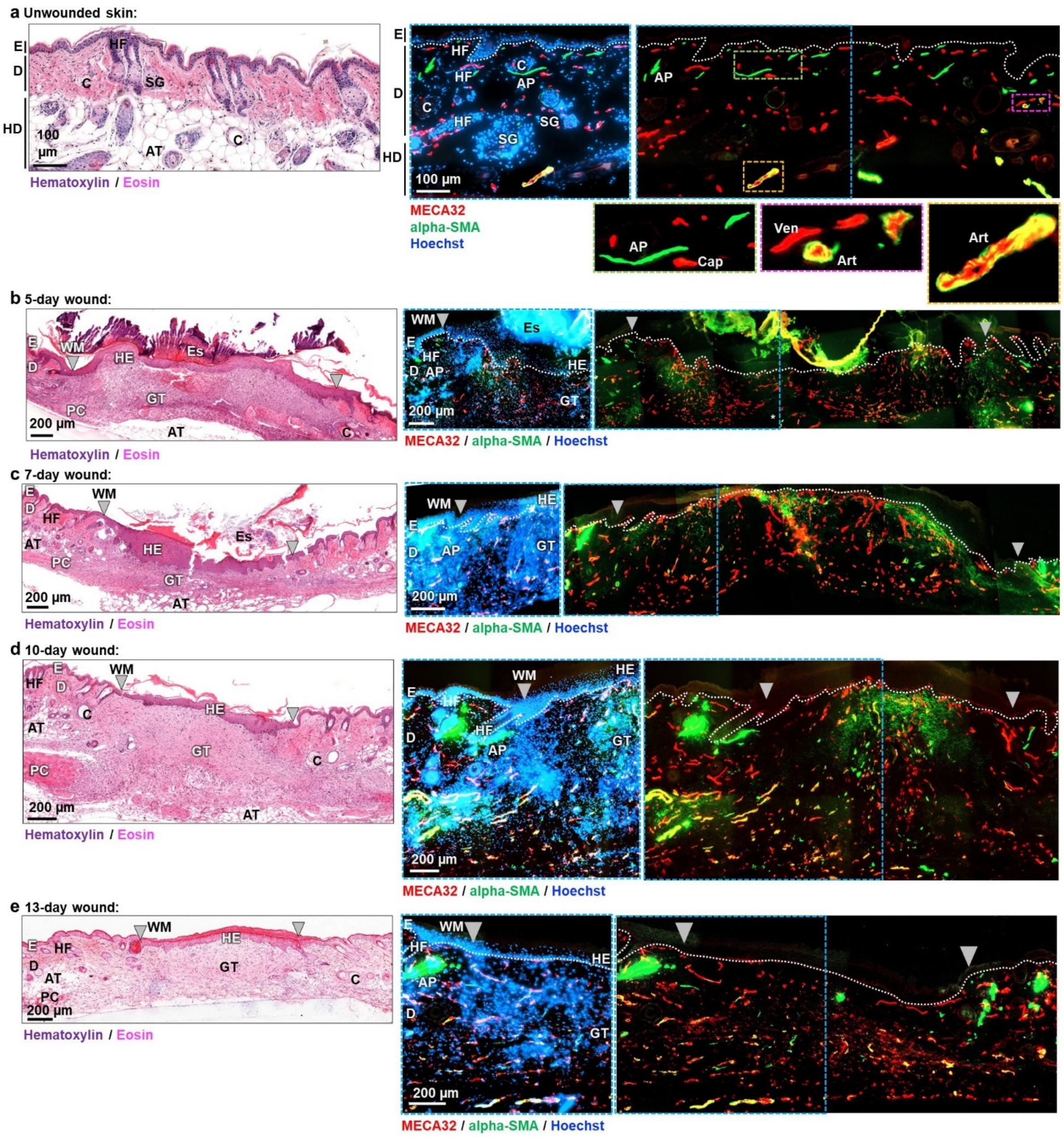

Figure S10. Overview of histological and immunofluorescence stainings of SKH-1 mouse skin and excisional wounds. Unwounded skin and wounds at different time-points post-injury were harvested, sectioned and stained using H&E (left) or immunostained with antibodies (right) against MECA32 (red) and alpha-SMA (green), with Hoechst nuclear counterstain (blue) and overlay with anatomical annotations (middle). **a** Unwounded skin, showing epidermis, dermis and hypodermis and associated appendages (left) and vasculature (right), including superficial capillaries (green box), deeper paired venules and arterioles (purple box), and larger arterioles of the hypodermis (orange box). **b** 5-day wound, showing the thickened hyperproliferative wound epidermis covered by a thick eschar and the underlying granulation tissue (left) and associated sprouting vasculature (right). **c** 7-day wound, showing the thickened hyperproliferative epidermis and underlying granulation tissue (left) and associated vasculature

(right). **d** 10-day wound, showing the thinning wound epidermis and underlying granulation tissue (left) and associated vasculature (right). **e** 13-day wound, showing the thin wound epidermis, and underlying maturing granulation tissue (left) and associated vasculature (right). White dotted line: epidermis-dermis/granulation tissue interface; E: Epidermis; D: Dermis; HD: Hypodermis; HF: Hair Follicle; SG: Sebaceous Gland; C: Cyst; AT: Adipose Tissue; PC: Panniculus carnosus; AP: Arrector Pili; Cap: Capillary; Ven: Venule; Art: Arteriole; WM, grey triangle: Wound Margin; Es: Eschar; HE: Hyperproliferative Epidermis; GT: Granulation Tissue

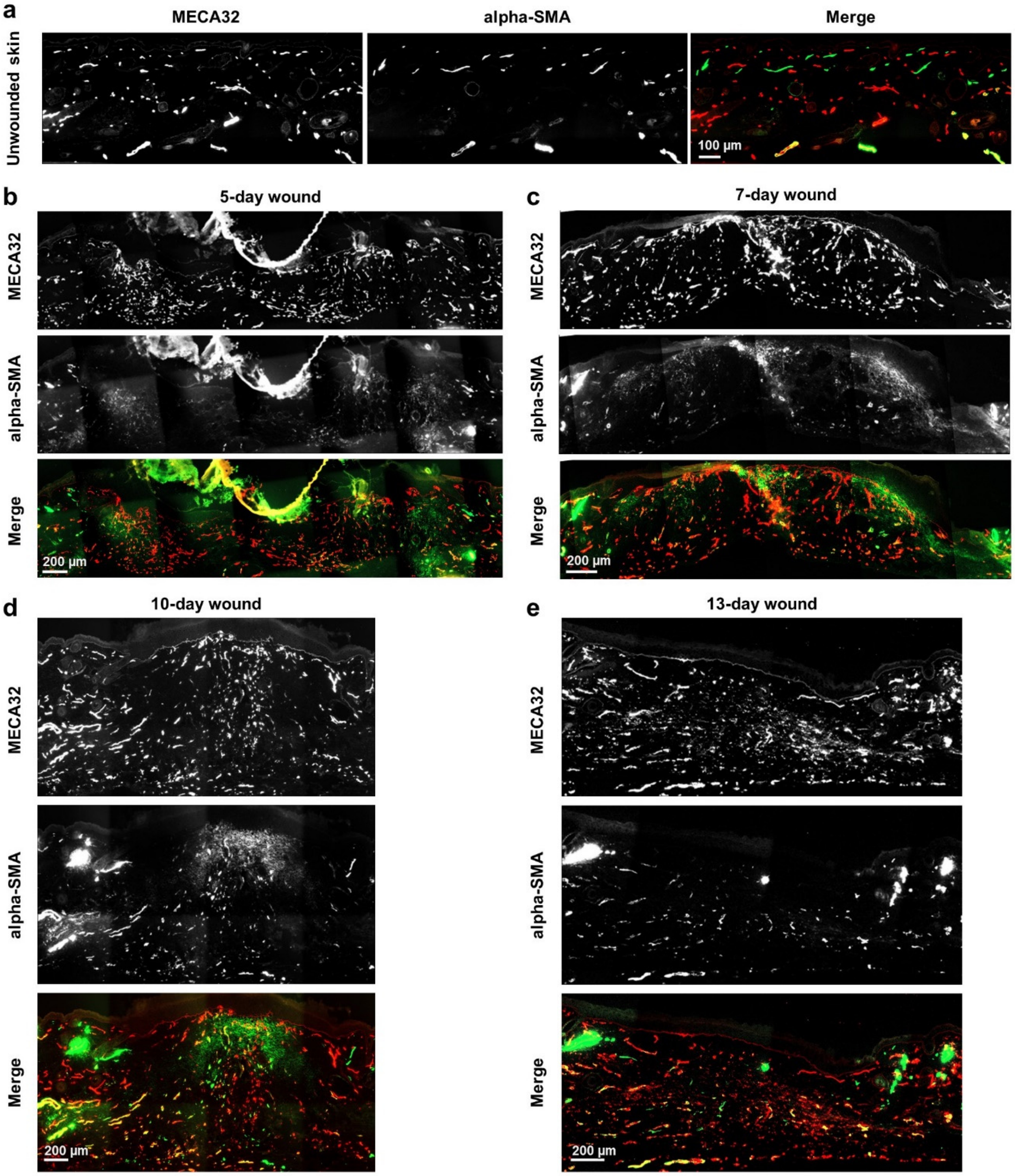

Figure S11. Detailed immunofluorescence analysis of SKH-1 mouse skin and excisional wounds. Panels as in Figure S10 with staining for MECA32 and alpha-SMA separated. **a** Unwounded skin. **b-e** Wounds at different time-points post-injury. See Figure S10 for details.

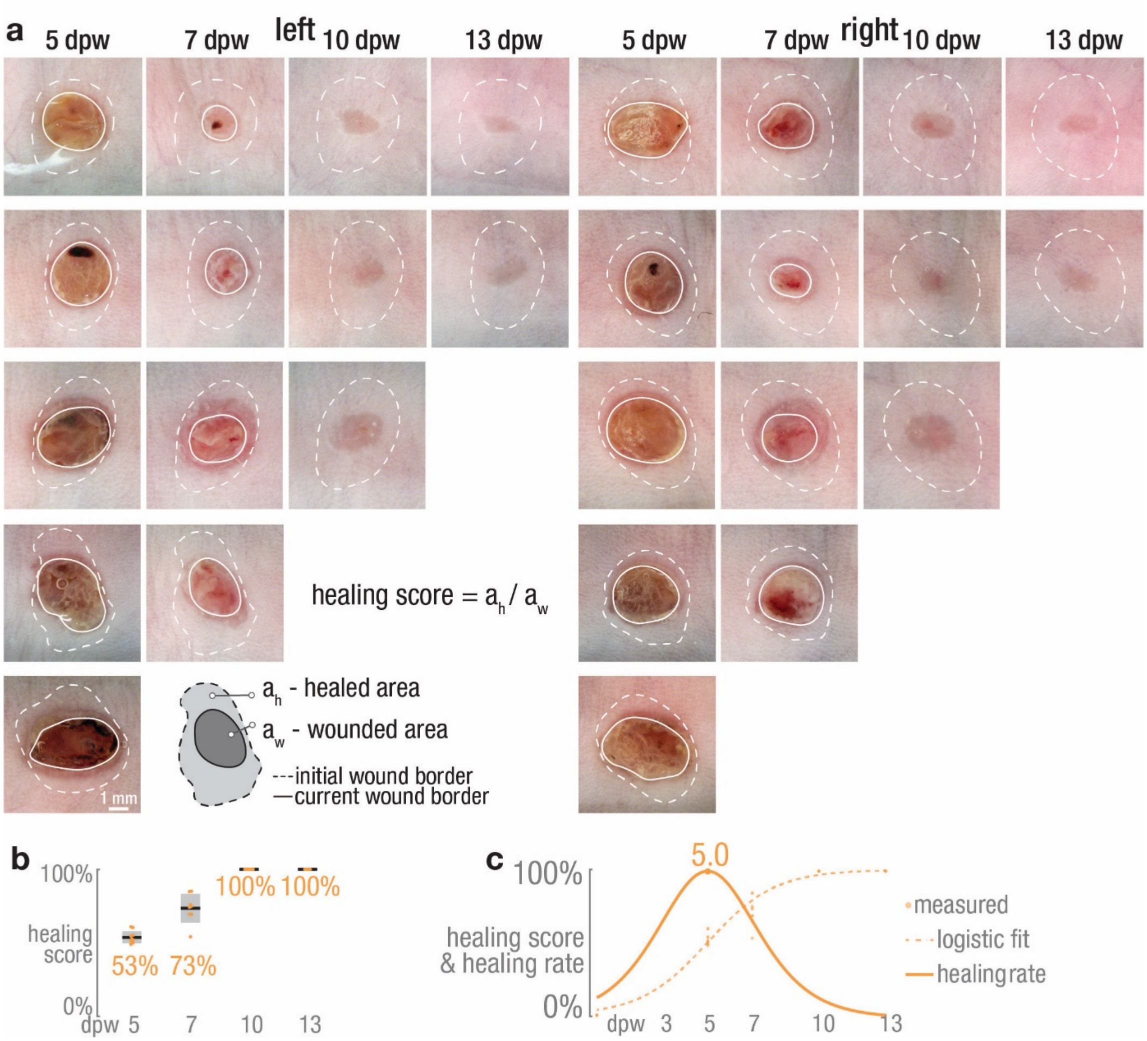

Figure S12. Quantification of wound healing based on photographs. a Manually masked wound areas for quantification of wound healing score. Dashed white lines – approximate location of initial wound border; solid white lines – apparent current wound border. Healing score defined as ratio of healed wound area vs. the current wound area in percent, see also insert. **b** Visualization of continuous wound closure with complete closure at 10 dpw. Orange dots represent individual wound data; black lines represent mean values; gray boxes represent 95% confidence interval. **c** Time-courses of wound healing (based on data in b) reveal fastest wound closure at 5.0 dpw. Orange dots - individual wound data; dashed lines - logistic fit; solid lines - normalized healing rates.

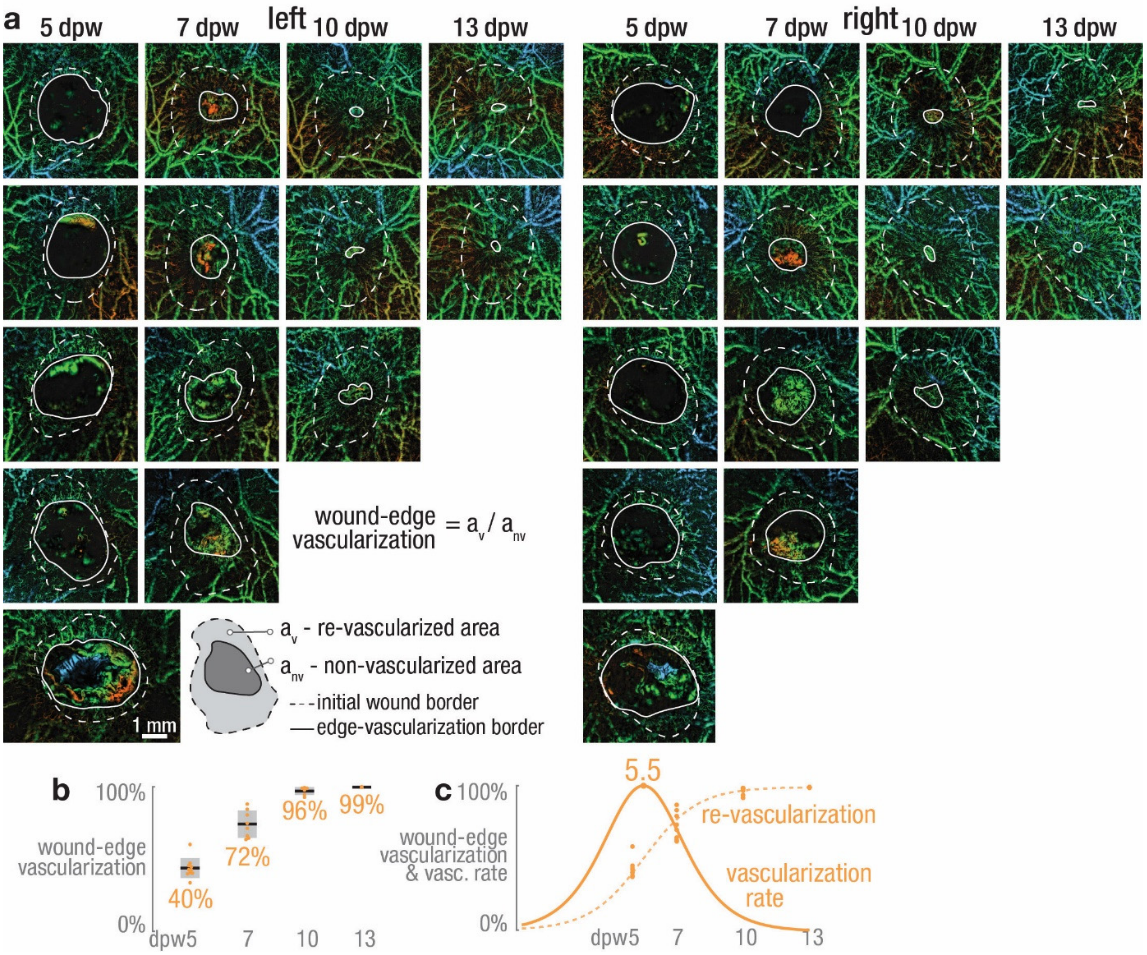

Figure S13. Quantification of wound-edge vascularization based on LSOM images. a Manually masked wound areas for quantification of wound vascularization based on volumetric LSOM projections. Dashed white lines – approximate location of initial wound border; solid white lines – apparent current wound border. Healing score defined as ratio of healed wound area vs. the current wound area in percent, see also insert. **b** Visualization of continuous wound-edge revascularization which is complete at 13 dpw. Orange dots represent individual wound data; black lines represent mean values; gray boxes represent 95% confidence interval. **c** Time-courses of revascularization (based on data in B) reveals strongest angiogenesis at 5.5 dpw, with a delay of only half a day following the wound closure (see Figure S11). Orange dots - individual wound data; dashed line - logistic fit; solid line - normalized vascularization rates.

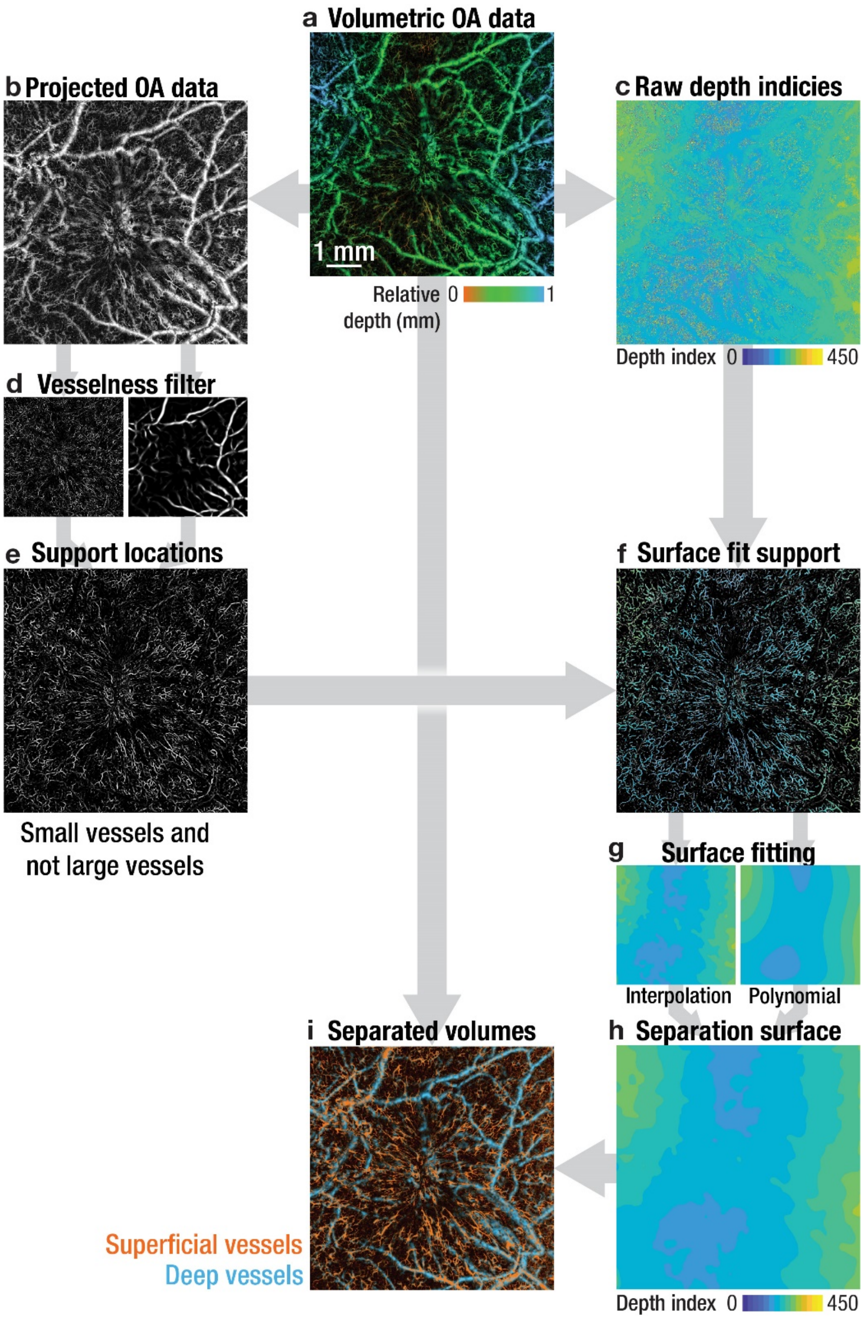

Figure S14. Surface detection algorithm for segmentation into volumes containing superficial and deep vessels. a Volumetric LSOM datasets. **b** Projection onto 2D maps. **c** Raw depth indices are also extracted from the volumetric LSOM data as location of the maximum signal. **d** The projected data is processed to enhance small and large vessels, respectively, using a vessel enhancement filter. **e** Areas that contain small but not large vessels are then used as support location for a surface fit. **f** Raw depth indices (panel c) and support locations are combined. **g** Support for surface fitting is then generated using either interpolation (left) or a surface polynomial (right). **h** An average of the two readings from the previous panel. **i** The original LSOM volume is separated into two volumes containing superficial and deep vessels.

Usage or figure panel	Scan size (mm x mm)	Step Size (µm)	Scan Time (min:sec)	Number of voxels[1]	Scan Rate (Voxels/s)
preview scans	11×30	50	0:18	1.13×10^7	6.29×10^5
2A, 2B, 3B - 3G[2]	11×30	10	6:57	2.83×10^8	6.78×10^5
preview scans	7×7	25	0:16	6.72×10^6	4.20×10^5
2C, 2D, 3B - 3G[3]	7×7	5	4:13	1.68×10^8	6.64×10^5
2E	3×2	2	3:46	1.29×10^8	5.69×10^5

Supplementary Table 1. Scan parameter overview. [1]Number of effective voxels, with x-y-size of voxels based on lateral step-size and z-size of 17.5 µm, i.e. half the 35 µm axial resolution according to the Nyquist–Shannon sampling theorem. [2]Large panels only. [3]Small panels only

Supplementary Video 1. Animation of assembly and operation of the DIM.

Supplementary Video 2. Operation of the LSOM system. Ease of operation, fast imaging speed and DIM assembly are illustrated using a dorsal-skin, *ex vivo* tissue sample.

Supplementary PDF 1. Interactive, 3D visualization of the DIM. (Open in latest version of Adobe Acrobat Reader for correct display and full functionality.)

TITLE	
PART NO	
REVISION	
DESIGNER	
ENGINEER	

NOTES

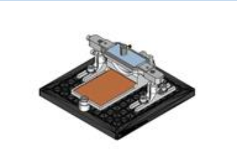
Default

THE INFORMATION AND/OR MATERIAL IN THIS DOCUMENT IS THE PROPERTY OF AND RESTRICTED INFORMATION AND/OR MATERIAL OF THE AUTHOR. THIS INFORMATION MAY NOT BE USED, REPRODUCED, PUBLISHED OR DISCLOSED TO OTHERS WITHOUT WRITTEN AUTHORIZATION. IT IS TO BE USED ONLY FOR MANUFACTURING ITEMS SPECIFIED WITHIN THE DOCUMENT.
